# Comparison Between Natural Products and Chlorhexidine in Non-Surgical Periodontal Therapy: A Systematic Review of Randomized Clinical Trials

**DOI:** 10.3390/dj14020110

**Published:** 2026-02-13

**Authors:** Andrea Scribante, Matteo Pellegrini, Maurizio Pascadopoli, Valentino Natoli, Valentina Poma, Andrea Butera

**Affiliations:** 1 Section of Dentistry, Department of Clinical, Surgical, Diagnostic and Pediatric Sciences, University of Pavia, 27100 Pavia, Italy; andrea.scribante@unipv.it (A.S.);; 2 Unit of Dental Hygiene, Section of Dentistry, Department of Clinical, Surgical, Diagnostic and Pediatric Sciences, University of Pavia, 27100 Pavia, Italy; valentina.poma01@universitadipavia.it (V.P.); andrea.butera@unipv.it (A.B.); 3 Department of Dentistry, School of Biomedical and Health Sciences, European University of Madrid, 28670 Madrid, Spain

**Keywords:** chlorhexidine, clinical parameters, dentistry, natural products, non-surgical periodontal therapy, periodontitis, systematic review

## Abstract

**Objectives**: To evaluate the clinical effectiveness and safety of natural products compared with chlorhexidine (CHX) as adjuncts to non-surgical periodontal therapy (NSPT) in patients with periodontitis. **Materials and Methods**: This systematic review was conducted in accordance with the PRISMA 2020 guidelines and registered in PROSPERO (CRD420251133219). Electronic searches of PubMed, Scopus, and Web of Science were performed to identify randomized controlled trials (RCTs) published between 2020 and 2025. Eligible studies included adult patients with periodontitis treated with NSPT, comparing CHX-based products with natural formulations (mouthwashes, gels, irrigants, or dentifrices). Data extraction included product type, concentration, mode of application, follow-up duration, and primary periodontal outcomes. Study quality was assessed using the NIH Quality Assessment Tool. Results: Thirteen randomized controlled clinical trials met the inclusion criteria. Natural products such as *Curcuma longa*, *Morus alba*, *Spirulina platensis*, *Propolis*, *Triphala*, and *Lycium barbarum* demonstrated improvements in clinical attachment level (CAL) and probing pocket depth (PPD) comparable to those obtained with CHX, along with significant reductions in bleeding on probing (BoP) and plaque index (PI). Probiotic- and ozone-based treatments also showed favorable clinical outcomes, with faster healing and fewer adverse effects, such as tooth staining and taste alteration. Follow-up periods ranged from 14 days to 3 months. **Conclusions:** Natural products appear to be safe and effective alternatives to CHX when used as adjuncts to non-surgical periodontal therapy, providing comparable clinical benefits with a lower incidence of side effects. Nevertheless, further large-scale, long-term randomized trials are needed to standardize formulations and concentrations and to confirm the durability of these clinical effects.

## 1. Introduction

Periodontitis is one of the most prevalent chronic inflammatory diseases of the oral cavity, characterized by the progressive destruction of the supporting tissues of the teeth, leading to clinical attachment loss and alveolar bone resorption [1]. The 2017 classification proposed by the American Academy of Periodontology (AAP) and the European Federation of Periodontology (EFP) introduced a staging and grading system that allows for a more accurate assessment of disease severity and progression rate, enabling a personalized therapeutic approach [2]. This classification also emphasized the systemic relevance of periodontitis, which has been associated with cardiovascular disease, diabetes mellitus, and pregnancy complications [3].

The first-line treatment remains non-surgical periodontal therapy (NSPT), which represents the cornerstone of periodontal care and includes both professional and patient-driven measures, such as detailed oral hygiene instruction, professional mechanical plaque removal, scaling, and root planing, all aimed at reducing the bacterial biofilm and halting disease progression [4].

As an adjunct to these procedures, chlorhexidine (CHX) has historically played an important role and is considered the gold standard among oral antiseptics due to its broad antimicrobial spectrum and its ability to bind to oral tissues, thereby maintaining a prolonged action (substantivity) [5]. However, its use is limited by well-documented side effects, including extrinsic tooth staining, taste alteration, mucosal desquamation, and increased supragingival calculus formation [6]. Moreover, in vitro studies have shown a negative impact on collagen synthesis by gingival fibroblasts, suggesting potential adverse effects on long-term healing [7]. For these reasons, clinical guidelines recommend its use only for limited periods and under specific conditions, such as postoperative phases or in cases of severe gingivitis [5].

Given these limitations, dental research has increasingly focused on natural products with antimicrobial and anti-inflammatory properties and a more favorable safety profile [8]. In recent years, several natural compounds—including triphala, spirulina, turmeric, *Piper betle* extract, and ozonized gels—have been investigated in clinical and experimental studies, showing promising results as adjuncts in NSPT and as potential alternatives to CHX [9,10,11,12,13,14]. These substances, in addition to reducing bacterial load and modulating the inflammatory response, appear to offer greater tolerability and a lower incidence of side effects, representing a therapeutic approach of growing interest in periodontology [15].

Despite the increasing interest in the use of natural products as adjuncts to NSPT, the available evidence remains limited, heterogeneous, and often methodologically weak. To date, there is no comprehensive and consistent understanding of whether—and to what extent—these compounds can serve as a clinically reliable alternative to CHX, which remains the gold standard for chemical plaque control. The few comparative clinical studies available show substantial differences in study design, small sample sizes, and short follow-up periods, making it difficult to assess the stability, durability, and long-term safety of their effects. Moreover, variations in formulation, concentration, and mode of application of natural products further complicate direct comparisons and hinder a clear evaluation of their true clinical effectiveness. Importantly, although several narrative and systematic reviews have addressed the use of herbal or natural agents in periodontal therapy, most have focused on gingivitis, plaque control, or general oral hygiene, or have included non-randomized and in vitro studies, without specifically comparing natural products with CHX as adjuncts to NSPT using standardized clinical periodontal outcomes.

In this context, the present article aims to conduct a systematic review to evaluate the effectiveness of natural products as adjuncts in non-surgical periodontal therapy, analyzing their main advantages and disadvantages in terms of clinical efficacy, safety, and tolerability, with particular attention to outcomes related to key periodontal parameters: Plaque Index (PI), Bleeding on Probing (BoP), Probing Pocket Depth (PPD), and Clinical Attachment Level (CAL).

## 2. Materials and Methods

### 2.1. Study Protocol and Registration

The current review protocol has been officially registered on the PROSPERO platform (CRD420251133219), available online: https://www.crd.york.ac.uk/PROSPERO/view/CRD420251133219 (accessed on 26 August 2025).

### 2.2. Focused Questions

In adolescents and adults with clinically diagnosed periodontitis of any stage and grade according to the 2017 AAP/EFP classification, are natural products used as adjuncts to NSPT clinically effective and safe compared with CHX-based formulations in improving periodontal clinical parameters (PI, BoP, PPD, and CAL)?

### 2.3. Search Strategy

A three-step search strategy was conducted in accordance with the methodology outlined by JBI for systematic reviews [16]. A systematic search of PubMed (MEDLINE), Scopus, and Web of Science (WoS) was independently performed by two reviewers (V.P. and M.P.). Initially, a preliminary search was carried out to identify relevant keywords and Medical Subject Headings (MeSH). These terms were subsequently refined and expanded by analyzing the titles, abstracts, and indexing terms of selected articles. In the final step, the reference lists of all included studies were manually screened to identify any additional eligible publications.

The research question was developed using the PICO framework, defining adolescents and adults with periodontitis as the study population; natural-based oral care products used as adjuncts to NSPT as the intervention; CHX-based formulations as the comparator; and variations in periodontal clinical parameters, namely PI, BoP, PPD, and CAL, together with safety and tolerability, as the outcomes of interest. Studies published up to 30 August 2025 were considered, with no restrictions on language or clinical setting. Gray literature was not included, as the objective of this review was to synthesize evidence from peer-reviewed clinical studies published in internationally indexed journals, thereby ensuring methodological rigor and reproducibility (Table 1).

As PubMed, Scopus, and WoS use different indexing systems, the search strategy was adapted accordingly. In PubMed, both MeSH terms and free-text keywords were applied, whereas in Scopus and WoS, equivalent free-text terms combined with Boolean operators (AND, OR) were used to ensure consistency. The complete search strategies, including MeSH terms (e.g., “Periodontitis,” “Mouthwashes,” “Chlorhexidine,” “Phytotherapy”) and an extensive set of free-text keywords covering specific natural compounds (e.g., propolis, curcumin, aloe, green tea, miswak, triphala, grape seed extract), are provided in Table S2 (Supplementary Materials), along with the number of records retrieved from each database.

All retrieved references were imported into EndNote (Clarivate Analytics, Philadelphia, PA, USA) to identify and remove duplicates, and the deduplicated dataset was subsequently uploaded into Covidence (Veritas Health Innovation, Melbourne, Australia) for the screening and selection process. Titles and abstracts were independently screened by two reviewers (V.P. and M.P.), and full-text articles of potentially eligible studies were assessed against the predefined inclusion criteria. Any disagreements were resolved through discussion and consensus with two additional reviewers (A.S. and A.B.).

This review adhered to the PRISMA 2020 guidelines [17], as summarized in Table S1 (Supplementary Materials).

### 2.4. Eligibility Criteria

This review was conducted based on predefined inclusion and exclusion criteria, summarized in Table 2. To ensure comparability of interventions and outcomes, only randomized controlled trials (parallel-group or split-mouth designs) conducted in humans with a clinical diagnosis of periodontitis were considered eligible. The target population included adolescents and adults diagnosed with periodontitis according to the 2017 AAP/EFP classification [2]. Only studies reporting periodontal outcomes measured with validated indices and standardized probing protocols were considered eligible. No restrictions were applied regarding language, clinical setting, or follow-up duration, and studies published up to 30 August 2025 were included. Given the objective of reflecting real-world clinical practice, no restrictions were applied regarding the specific type of natural product or CHX concentration, provided that both were used as adjuncts to NSPT and compared within the same randomized trial. However, only studies reporting detailed information on formulation, concentration, and mode of administration were included, allowing structured comparison and assessment of clinical heterogeneity.

The primary objective was to compare the clinical effects of natural-based products and CHX as adjuncts to NSPT on key periodontal parameters: PI [18], BoP [19], PPD [20], and CAL [21]. Secondary outcomes included safety, tolerability, and patient-reported measures. When available, data on oral microbiota modulation were also collected and synthesized narratively, without influencing the focus on primary outcomes.

### 2.5. Study Selection Process

The study selection process was conducted in two phases using Covidence (Veritas Health Innovation, Melbourne, Australia). The search was initiated on 10 June 2025 and completed on 30 August 2025. In the first phase, titles and abstracts were screened to exclude irrelevant studies. In the second phase, the full texts of potentially relevant articles were reviewed in detail to confirm eligibility according to the predefined inclusion and exclusion criteria (Table 2).

Disagreements between the two primary reviewers (V.P. and M.P.) were resolved through discussion and consensus with two additional reviewers (A.S. and A.B.). The selection process, along with the reasons for exclusion at the full-text review stage, is presented in Table S3 (Supplementary Materials).

### 2.6. Data Extraction

After the screening process, the most relevant data from each included study were extracted using a customized data extraction form developed in Microsoft Excel. The form was designed in advance by the two lead authors (V.P. and M.P.) and pilot-tested on the first five eligible articles to ensure consistency and clarity. Any necessary adjustments were made in consultation with the senior reviewers (A.S. and A.B.) and duly documented.

Data extraction was independently performed by V.P. and M.P., and the accuracy of the extracted information was verified through cross-checking. Discrepancies were resolved through discussion and, when required, with the involvement of A.S. and A.B.

Extracted information included study characteristics (authors, year, country, study design); population features (sample size, diagnostic criteria for periodontitis); intervention details (type of natural product, formulation, dosage, frequency, duration); comparator characteristics (CHX-based product, formulation, dosage, duration); and outcomes of interest. The primary outcomes were changes in periodontal clinical parameters—PI, BoP, PPD, and CAL. Secondary outcomes included safety, tolerability, and patient-reported measures. To ensure methodological consistency and clinical validity, only studies using standardized and validated periodontal indices and measurement protocols were considered eligible. Specifically, plaque accumulation was required to be assessed using validated plaque indices, primarily the Silness and Löe PI [22], or other standardized site-based indices with comparable scoring systems, such as the Plaque Control Record (PCR) [23] or the Turesky modification of the Quigley–Hein Index [24]. Bleeding outcomes were considered eligible when recorded using established bleeding indices, including BoP [25], Sulcus Bleeding Index (SBI) [26], Papillary Bleeding Index (PBI) [26], or Gingival Bleeding Index (GBI) [25], all of which assess gingival inflammatory response following periodontal probing using standardized criteria. PPD [21] and CAL [21] were required to be measured in millimeters using calibrated periodontal probes and standardized probing techniques, as described in the original trials. Only studies clearly reporting the periodontal assessment methods and scoring systems were included. A summary of the standardized indices and scoring criteria accepted in the present review is provided in Table 3.

To further enhance reproducibility, intervention details were reported according to the Template for Intervention Description and Replication (TIDieR) checklist, including rationale, materials, mode of administration, procedures in both intervention and control groups, setting, timing and duration, and adherence.

### 2.7. Quality Assessment

The methodological quality of the included randomized controlled trials was evaluated using the National Heart, Lung, and Blood Institute (NHLBI) Quality Assessment Tool for Controlled Intervention Studies [27]. This checklist allowed for a structured appraisal of key aspects such as clarity of objectives, adequacy of sample size, description of randomization and blinding, handling of withdrawals and dropouts, and appropriateness of statistical analyses.

In parallel, the risk of bias (RoB) was assessed using the Cochrane Risk of Bias 2.0 (RoB 2.0) tool [28], which specifically addresses potential biases across five domains: randomization process, deviations from intended interventions, missing outcome data, measurement of the outcome, and selection of the reported result. Each domain was judged as “low risk of bias,” “some concerns,” or “high risk of bias,” and an overall judgment was assigned to each study.

Both the NHLBI and RoB 2.0 assessments were independently conducted by two reviewers (V.P. and M.P.). Disagreements were resolved through discussion and, when necessary, with the involvement of two additional reviewers (A.S. and A.B.).

The outcomes of the quality appraisal (NHLBI) and risk of bias analysis (RoB 2.0) are presented in Tables S4 and S5 (Supplementary Materials), in accordance with PRISMA 2020 recommendations [28], to ensure transparency and reproducibility. Additionally, Table S6 (Supplementary Materials) reports the results of the NHLBI Quality Assessment Tool for Controlled Intervention Studies.

### 2.8. Data Synthesis and Analysis

The results of the included studies were synthesized using a narrative and descriptive approach, in accordance with PRISMA 2020 recommendations, given the substantial clinical and methodological heterogeneity observed. A meta-analysis was not conducted, as pre-specified in the protocol, because of differences in study designs, natural product formulations, treatment protocols, inclusion criteria, outcome definitions, and follow-up durations, which made statistical pooling inappropriate and potentially misleading. This heterogeneity was considered intrinsic to the clinical question, as natural products represent a heterogeneous therapeutic class. For this reason, data were stratified and interpreted according to type of natural compound, formulation, and CHX concentration, rather than pooled indiscriminately.

Data were extracted and organized into structured summary tables to facilitate qualitative comparison between natural products and CHX. The synthesis focused on the main periodontal clinical parameters (PI, BoP, PPD, and CAL), while secondary outcomes included safety, tolerability, and patient-reported measures. Reported findings were stratified according to the type of intervention (natural products vs. CHX) and, where available, compared with conventional non-pharmacological therapies.

Follow-up times ranged from a few weeks to several months, contributing to variability in the results and limiting direct comparability across studies. Some trials reported quantitative improvements in clinical parameters, whereas others emphasized patient-centered outcomes such as tolerability and acceptability, often without predefined thresholds for clinical success.

The completeness of intervention reporting was also evaluated using the TIDieR checklist, to ensure standardized and transparent assessment of intervention characteristics across studies.

## 3. Results

The electronic search of PubMed, Scopus, and Web of Science identified a total of 3330 records. After removal of duplicate records (n = 83), 3247 records were screened at the title and abstract level. At this stage, 3047 records were excluded because they did not meet the eligibility criteria. Specifically, excluded records comprised 1256 non-human or in vitro studies, 353 non-primary publications (reviews, letters, and editorials), 345 non-randomized studies or studies not classifiable as randomized clinical trials, 379 studies not related to the dental field, and 714 studies not related to oral medicine or oral surgery.

Following this screening process, 200 reports were sought for retrieval and all were successfully retrieved. These 200 full-text articles were assessed for eligibility. After full-text evaluation, 187 articles were excluded for the following reasons: lack of comparison with chlorhexidine (n = 27), study populations not affected by periodontitis (n = 41; including gingivitis, plaque control, halitosis, and mucositis), systemic or non-dental interventions (n = 21), interventions not relevant to the research question (n = 40), and outcomes not relevant to the present review (n = 58), including surgical, endoscopic, or endodontic treatments.

**Figure 1:**
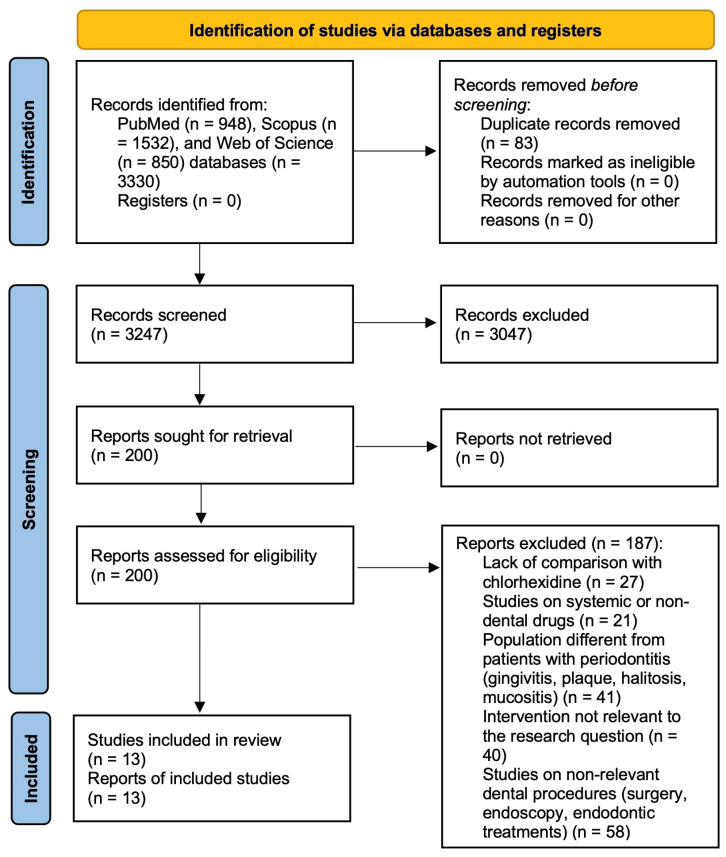
PRISMA 2020 flow diagram of the study selection process.

Ultimately, 13 randomized controlled trials fulfilled all eligibility criteria and were included in the final qualitative synthesis [10,11,12,13,14,29,30,31,32,33,34,35,36]. The study selection process is illustrated in Figure 1 (PRISMA 2020 flow diagram), and the complete list of excluded full-text articles with detailed reasons for exclusion is reported in Table S3 (Supplementary Materials).

### 3.1. Risk of Bias Assessment

RoB was assessed using the RoB 2 tool (Cochrane Collaboration [28]), which evaluates five domains: (D1) randomization process, (D2) deviations from intended interventions, (D3) missing outcome data, (D4) measurement of the outcome, and (D5) selection of the reported result. The evaluation was independently conducted by two blinded reviewers (V.P. and M.P.) to ensure accuracy and reliability.

Most studies were rated as presenting some concerns or high risk of bias, mainly in domains D2 and D5. In contrast, two studies—Dolly (2024) [11] and Scribante (2024) [14]—were judged to have a low risk of bias across all domains, reflecting rigorous methodological conduct and transparent reporting.

Studies by Agarwal (2020) [31], Rathod (2023) [10], Basudan (2023) [35], Waqar (2024) [32], Chawla (2024) [34], Gunjal (2024) [29], Seth (2022) [33], Siddharth (2020) [30], Guru (2020) [12], Amee (2023) [36], and Sundaram (2021) [13] were classified as having some concerns. While these studies appropriately handled missing data (D3) and outcome measurement (D4), several displayed limitations in D2 (deviations from intended interventions) and D5 (selection of the reported result).

Specifically, Gunjal (2024) [29], Waqar (2024) [32], Seth (2022) [33], Guru (2020) [12], and Chawla (2024) [34] provided incomplete documentation on blinding procedures or handling of protocol deviations, resulting in moderate risk within D2. Similarly, Sundaram (2021) [13], Seth (2022) [33], Guru (2020) [12], Basudan (2023) [35], and Amee (2023) [36] raised concerns in D5 due to potential selective reporting or limited outcome description.

Detailed RoB judgments are provided in Supplementary Tables S4–S6. Table 4 summarizes the overall risk-of-bias assessment, Table 5 reports the baseline clinical characteristics and periodontal outcomes, while Table 6 provides a comprehensive overview of study design, intervention characteristics (type, concentration, and mode of application), and the comparative clinical effectiveness of natural products versus CHX across all included trials.

### 3.2. Results of Syntheses

Across the included studies, both CHX and natural products used as adjuncts to NSPT demonstrated significant improvements in PI, BoP, PPD, and CAL. Most trials reported comparable overall clinical efficacy between natural compounds and CHX, with some natural agents showing slightly superior outcomes in specific parameters (notably *Morus alba*, propolis, and curcumin-based formulations).

#### 3.2.1. Anti-Dental Plaque and Antibacterial Effects

Plant-based formulations such as *Morus alba* gel [29], *Matricaria chamomilla* mouthwash [31], and *Piper betle* extract rinse [13] achieved reductions in plaque accumulation, gingival inflammation, and microbial load similar to CHX. Notably, *Morus alba* produced a greater decrease in PPD, suggesting a potential pocket-reducing or regenerative effect.

Polyphenol-rich agents such as propolis [32,33] also demonstrated strong antibacterial activity. Both subgingival irrigation and mouthwash applications resulted in reductions in PI, GI, and PPD comparable to CHX, with some studies reporting greater decreases in BoP. *Spirulina platensis* [11] showed marked antibacterial activity against *Porphyromonas gingivalis*, while *Salvadora persica* [35] was equally effective in improving periodontal indices among non-smokers, although CHX remained superior in reducing *Candida albicans* carriage.

#### 3.2.2. Anti-Inflammatory Effects

Many of the evaluated natural products exhibited pronounced anti-inflammatory properties. Propolis [32,33] and curcumin-based formulations [12,30] significantly reduced gingival inflammation, BoP, and periodontal pocket depth, achieving outcomes comparable to or better than CHX. Chamomile (*Matricaria chamomilla*) [31] and *Piper betle* [13] also contributed to effective control of gingival inflammation, supporting their role as suitable adjuncts during the healing and maintenance phases of NSPT.

#### 3.2.3. Antioxidant and Host-Modulating Effects

Herbal compounds with antioxidant activity—including curcumin [12,30], Triphala [10], and *Lycium barbarum* [36]—produced consistent clinical and microbiological improvements. Curcumin gels, particularly when delivered via nanocarriers, achieved significant CAL gain and PPD reduction. *Lycium barbarum* uniquely increased salivary antioxidant levels, indicating an additional systemic benefit. Triphala, a polyherbal formulation rich in antioxidant and antimicrobial constituents, proved particularly beneficial in diabetic patients.

#### 3.2.4. Novel Biological and Oxidative Therapies

Innovative alternatives such as ozone gels [14] and probiotic mouthwashes [34] achieved outcomes similar to CHX with improved tolerability. Ozone therapy reduced plaque and bleeding without adverse effects, while probiotics promoted sustained microbial balance and a more stable reduction in pathogenic bacterial load.

#### 3.2.5. Overall Synthesis

Overall, the evidence indicates that natural products can achieve clinical outcomes comparable to CHX in improving periodontal health. Their favorable safety profiles and absence of common adverse effects—such as tooth staining, mucosal irritation, or taste alteration—make them promising options for long-term maintenance within NSPT.

## 4. Discussion

The analysis of the randomized controlled trials included in this review provides a coherent and integrated picture of the role of natural products as adjuncts to NSPT. In the selected studies, CHX, which has traditionally been considered the reference standard for chemical plaque control, was compared with a broad spectrum of natural agents. These included herbal extracts such as *Curcuma longa*, *Matricaria chamomilla*, propolis, Triphala, *Morus alba*, *Piper betle*, *Lycium barbarum* and *Salvadora persica* [9,10,11,12,13,14], as well as biologically active compounds like Spirulina platensis and probiotics, and oxidizing agents such as ozone [37,38,39,40,41,42,43,44,45,46,47]. Across the trials, subgingival local drug-delivery systems, including gels, nanocarriers and pocket irrigation devices, showed stronger antibacterial, anti-inflammatory and antioxidant effects than mouthrinses, a finding that can be attributed to their ability to achieve higher intra-pocket concentrations, to provide sustained release, and to penetrate more effectively into inflamed periodontal tissues.

Taken together, the available evidence consistently indicates that natural products are able to produce significant improvements in the main clinical parameters of periodontal disease. These include reductions in PI, gingival inflammation, BoP and PPD, as well as gains in clinical attachment level. In most of the trials, the magnitude of these improvements was comparable to that observed with CHX, and intergroup differences only rarely reached statistical significance. In some cases, such as with *Morus alba*, propolis and Triphala, natural agents even produced superior results for specific clinical outcomes. These findings, however, must be interpreted cautiously. They derive from heterogeneous studies that were generally small in size, of short duration, and characterized by variability in diagnostic criteria, outcome definitions and intervention protocols. Such methodological diversity inevitably limits both the external validity and the generalizability of the reported effects.

Despite their chemical diversity, the natural products investigated share a set of biological mechanisms that can account for their clinical performance. Most of these agents display three key therapeutic properties: broad-spectrum antimicrobial activity against periodontal pathogens, the ability to modulate the host inflammatory response, and antioxidant effects that reduce oxidative tissue damage. While CHX acts mainly through non-selective disruption of bacterial cell membranes, natural compounds exert a more complex and multimodal action that combines microbial suppression with host-directed effects. At the molecular level, this activity is driven by well-defined bioactive constituents. Curcumin from *Curcuma longa*, polyphenols and flavonoids present in propolis, *Morus alba* and *Matricaria chamomilla*, tannins and gallic acid derivatives in Triphala, alkaloids and phenolic compounds in *Piper betle*, and polysaccharides and phycocyanins in *Spirulina platensis* all inhibit major periodontal pathogens while simultaneously down-regulating pro-inflammatory mediators such as TNF-α, IL-1β and matrix metalloproteinases. The combined antimicrobial and anti-inflammatory effects of these compounds are directly reflected in the reductions in PPD, BoP and attachment loss observed across the trials. In addition, unlike CHX, these natural agents also provide antioxidant protection, scavenging reactive oxygen species and preserving fibroblast function and collagen integrity, which supports wound healing and the stability of periodontal attachment. This shared biological profile explains why chemically diverse natural products tend to achieve clinical outcomes that fall within the same therapeutic range as CHX, even when delivered through different formulations and vehicles. Nonetheless, this apparent convergence should be viewed as a trend emerging from heterogeneous data rather than as definitive proof of true clinical equivalence across different clinical settings or patient populations.

**Figure 2:**
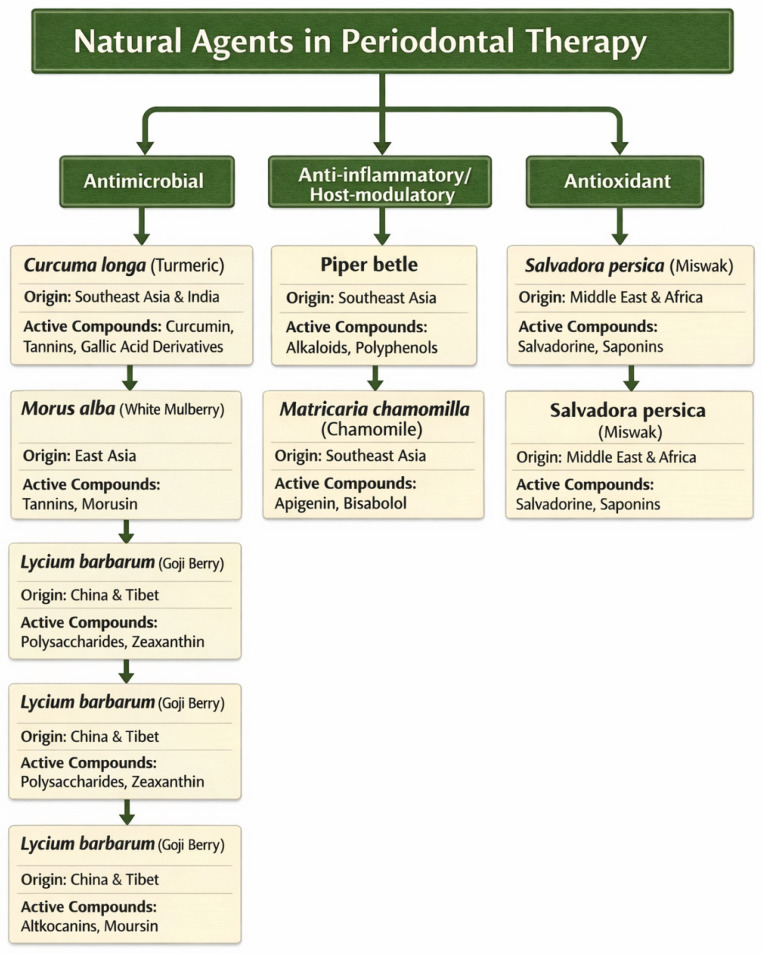
Botanical origin and principal bioactive compounds of the main plant-derived agents evaluated in this review.

The botanical origin, principal active constituents and shared biological properties of the main plant-based agents evaluated in this review are summarized in Figure 2.

*Curcuma longa* (curcumin) consistently exhibited potent anti-inflammatory and antioxidant properties through the modulation of cytokines and matrix metalloproteinases, resulting in PPD reduction and CAL gain comparable to CHX [12,30]. Advanced delivery systems, including nanocarriers, have further enhanced curcumin’s bioavailability and clinical performance. Propolis, rich in flavonoids and phenolic acids, showed similar improvements in plaque and inflammation indices compared with CHX [32,33], with the additional advantage of greater patient acceptance and minimal adverse reactions.

The polyherbal formulation Triphala displayed strong antibacterial and antioxidant activities, particularly beneficial in systemically compromised patients such as those with diabetes, where oxidative stress modulation is crucial [10]. *Spirulina platensis*, a protein- and antioxidant-rich microalga, produced significant reductions in PPD and CAL improvements [11], consistent with its regenerative and immunomodulatory potential. Likewise, *Matricaria chamomilla* demonstrated soothing and antimicrobial effects, confirming its role as a valuable maintenance-phase mouthwash [31].

Less-documented natural extracts such as *Morus alba*, *Piper betle*, and *Lycium barbarum* also yielded encouraging results [13,29,36], suggesting promising directions for the exploration of novel plant-derived bioactives. *Salvadora persica* (Miswak) was found to be clinically effective and well tolerated [35], although its antimicrobial spectrum appeared less pronounced than that of CHX, particularly against *Candida* species. Ozone-based gels [14] achieved outcomes comparable to CHX while avoiding tooth staining and taste alteration. Probiotic formulations [34] operated through a distinct biological pathway, promoting restoration of the oral microbial equilibrium rather than relying on direct bactericidal activity, in line with current concepts of ecological periodontal therapy. Importantly, this trend reflects short-term responses observed under controlled trial conditions and should not be extrapolated to long-term disease control or to all forms of periodontitis without further high-quality evidence.

Collectively, these findings highlight a clear trend toward the integration of natural and biologically derived substances in periodontal management. Natural products should no longer be regarded merely as marginal adjuncts, but as promising therapeutic options that may complement CHX in specific clinical contexts, particularly where tolerability and long-term use are relevant. While CHX remains useful in acute or high-inflammatory phases, natural agents offer superior tolerability and safety for chronic use, aligning with patient demand for biocompatible and sustainable oral care solutions. To facilitate interpretation of the heterogeneity observed across the included trials, the investigated products were classified using two complementary dimensions: their mode of administration, distinguishing mouthrinses from locally delivered subgingival formulations, and their predominant biological mechanism, defined as mainly antimicrobial, anti-inflammatory or host-modulating, antioxidant, or microbiome-modulating. This conceptual framework enables a more clinically relevant comparison among studies and makes it possible to identify trends that would not emerge from simple head-to-head product comparisons. When analyzed within this structure, locally delivered systems, including gels, nanocarriers and subgingival irrigants, proved to be the most consistently effective in reducing PPD and improving CAL, whereas mouthwashes mainly influenced plaque accumulation and gingival inflammation. In parallel, compounds rich in polyphenols and flavonoids, such as curcumin, propolis, chamomile and Triphala, exhibited the most pronounced anti-inflammatory and antioxidant activity, resulting in greater reductions in bleeding and soft-tissue inflammation. By contrast, microbiome-targeted approaches, including probiotics and Spirulina, were associated with a more stable suppression of periodontal pathogens while preserving the commensal oral flora.

To account for the heterogeneity of the interventions evaluated, the evidence from the included trials was interpreted by grouping products according to their category and delivery system rather than treating all natural agents as a single, uniform class. When analyzed in this way, clear and clinically relevant patterns become evident. Herbal and phytochemical compounds administered as mouthrinses or gels, including *Curcuma longa*, *Matricaria chamomilla*, *Piper betle*, *Morus alba*, *Lycium barbarum* and *Salvadora persica*, consistently produced reductions in plaque indices, gingival inflammation and BoP that were comparable to those obtained with CHX [13,29,30,31,35,36]. Within this group, formulations based on curcumin, especially when delivered through advanced vehicles such as nanocarriers, showed the most reproducible effects on PPD and CAL, in line with their strong anti-inflammatory and matrix-regulating activity [12,30]. Chamomile and *Piper betle* were particularly effective in controlling gingival inflammation [13,31], while *Salvadora persica* demonstrated clinical equivalence to CHX but a more limited antimicrobial spectrum, particularly against *Candida* species [35].

Polyherbal and resin-derived products, such as Triphala and propolis, displayed broader biological activity, combining antibacterial, antioxidant and host-modulating effects. In patients with diabetes, Triphala achieved periodontal improvements that were at least equivalent to those observed with CHX, underscoring the importance of oxidative stress modulation in systemically compromised individuals [10]. Propolis was associated with superior control of BoP and inflammatory indices in some trials, together with very good tolerability [32,33]. Biologically active and microbiome-focused agents, including *Spirulina platensis* and probiotic formulations, acted through mechanisms that differed from those of CHX. Rather than relying mainly on bactericidal activity, these products promoted immunomodulation, antioxidant defenses and microbial rebalancing, leading to reductions in probing depth and inflammation comparable to CHX while better preserving the physiological oral ecosystem [11,34].

Oxidative and physico-chemical agents, particularly ozone-based gels, achieved clinical and microbiological outcomes similar to those of CHX but with a more favorable safety and tolerability profile, notably avoiding tooth staining and taste alterations [14]. Across product categories, local drug-delivery systems, including subgingival gels and nanocarrier-based formulations such as nanocurcumin, spirulina gel, CHX gel and ozone gel, consistently produced larger and more sustained reductions in probing depth and greater gains in CAL than mouthwash-based approaches, regardless of the active compound employed. This finding indicates that the delivery system is a major determinant of clinical efficacy, alongside the intrinsic pharmacological properties of the agent [11,12,14,30].

Overall, this stratified interpretation shows that the apparent equivalence between natural products and CHX does not result from indiscriminate pooling of heterogeneous interventions, but from convergent evidence across multiple biologically distinct categories. Although individual agents differ in antimicrobial spectrum, host-modulating potential and delivery kinetics, their effects on the principal periodontal outcomes evaluated in these trials consistently fall within the same therapeutic range as CHX. Natural products should therefore be regarded not as a single interchangeable class, but as a group of adjunctive therapies whose optimal use can be tailored to patient characteristics, disease severity and the intended duration of treatment.

Although the overall findings are encouraging, several methodological and intrinsic constraints limit the robustness of the evidence base. Many of the included trials were characterized by small sample sizes (often fewer than 50 participants) and short follow-up periods (15–90 days), allowing the assessment of short-term effects but not of long-term stability. In addition, incomplete reporting of randomization procedures, blinding, sample size calculations, and the absence of intention-to-treat analyses increase the risk of bias and weaken the overall strength of inference.

These methodological limitations are mirrored in the risk-of-bias assessment performed with the Cochrane RoB 2.0 tool. Only two studies were classified as having a low risk of bias, while the majority were judged to present some concerns, mainly attributable to domains D2, which addresses deviations from intended interventions, and D5, which relates to selective reporting of results. Such ratings should not be interpreted as evidence of systematic flaws or invalid data, but rather as reflecting incomplete reporting of key methodological aspects, including adherence to the assigned interventions, blinding procedures, and the existence of prespecified statistical analysis plans. This issue is particularly common in small, single-center periodontal trials. More specifically, the absence of clearly stated intention-to-treat analyses, relevant to D2, and the lack of publicly accessible protocols or trial registrations, relevant to D5, introduce uncertainty regarding the consistency and transparency of outcome reporting. These potential sources of bias mainly affect the level of confidence in the estimated effects rather than their overall direction. Because most studies reported concordant findings indicating equivalence or non-inferiority of natural products compared with CHX across multiple independent outcomes, including PPD, CAL, PI and BoP, it is unlikely that the overall conclusions are driven solely by selective reporting. Nonetheless, the predominance of ratings indicating some concerns calls for cautious interpretation of effect sizes and does not support strong claims of superiority, instead justifying a moderate level of certainty. A further contributor to clinical heterogeneity is that not all of the included trials applied the 2018 World Workshop (AAP/EFP) classification for periodontitis and peri-implant diseases. Several investigations relied on earlier diagnostic systems, which may have influenced disease definitions, severity grading and patient selection, thereby reducing the comparability of their results both across studies and with current clinical standards. In addition, BoP, one of the earliest and most sensitive markers of periodontal inflammation and of disease recurrence after therapy, was not uniformly reported among the trials. The limited availability of BoP data further constrained the assessment of inflammatory control and weakened the ability to conduct robust comparisons between studies.

Incomplete reporting further limits the interpretability of the available evidence. As shown in Table 4 and Table 5, several important demographic, methodological and outcome-related variables, including age range, exclusion criteria, bleeding indices and clinical attachment level, were not consistently reported in the original articles and therefore had to be classified as not reported. Although the full texts were examined in detail to retrieve missing information whenever possible, a number of studies, particularly those investigating propolis, probiotics, *Salvadora persica* and *Lycium barbarum*, either did not provide these data or presented them in formats that did not allow reliable extraction. The absence of standardized and comprehensive reporting restricts the characterization of the study populations, impairs evaluation of baseline comparability and limits more refined comparisons across trials, thereby increasing uncertainty and reducing the precision of the estimated effects. These deficiencies reflect a broader weakness of the periodontal literature and underscore the need for stricter compliance with CONSORT reporting guidelines in future trials of natural adjuncts to non-surgical periodontal therapy.

Limitations of the literature search strategy must also be acknowledged. Although PubMed, Scopus and Web of Science together cover a substantial proportion of peer-reviewed biomedical and dental journals, the exclusion of databases such as EMBASE, the Cochrane Library and CINAHL may have led to the omission of some relevant studies. In addition, the intentional exclusion of gray literature, including theses, conference abstracts and other non-peer-reviewed sources, increases the likelihood of publication bias, since studies reporting negative or inconclusive results are less likely to be published in indexed journals. Consequently, the body of evidence synthesized in this review may overestimate the true clinical effectiveness of natural products when compared with CHX.

Additional uncertainty arises from the lack of standardization across natural product formulations. Extract concentrations (ranging from 1% to 25%) and delivery vehicles (mouthwashes, gels, irrigants, or subgingival systems) varied widely, limiting comparability and reproducibility. Furthermore, the intrinsic variability of plant-derived materials—driven by geographic origin, seasonal factors, and extraction methods—affects chemical composition and biological activity. Some compounds, such as curcumin, also exhibit poor solubility and bioavailability, which may reduce in vivo efficacy compared with in vitro expectations. Although these agents generally display favorable safety profiles, they are not entirely free from adverse effects, including mucosal irritation, taste alteration, or local hypersensitivity reactions, and occasional interactions with systemic medications cannot be excluded. Taken together, these methodological limitations, combined with the likelihood of publication bias, lower the overall certainty of the evidence and reinforce the need for standardized protocols and high-quality randomized controlled trials in this field.

Future investigations should prioritize the development of standardized formulations with well-defined concentrations and delivery systems, together with multicenter, large-scale randomized trials featuring extended follow-up to confirm long-term efficacy and safety compared with CHX. The exploration of novel controlled-release systems, such as gels or intra-pocket devices, could further enhance the therapeutic performance of these natural agents. Moreover, evaluating the ecological effects of natural and probiotic formulations on the oral microbiota represents a promising research direction.

## 5. Conclusions

This systematic review shows that a wide range of natural and biologically derived adjuncts to non-surgical periodontal therapy, including phytochemical agents, polyherbal formulations, biologically active compounds and oxidative products, are capable of producing clinically relevant short-term improvements in PI, BoP, PPD and CAL that are broadly comparable to those achieved with CHX in the settings examined. These effects are mediated through complementary mechanisms, encompassing antimicrobial activity, modulation of the host inflammatory response, antioxidant action and rebalancing of the oral microbiome. At the same time, the considerable clinical and methodological heterogeneity of the available trials restricts the strength, external validity and overall certainty of these comparisons.

When appropriately aligned with their mode of administration, particularly in the context of local drug-delivery systems, agents such as *Curcuma longa*–based formulations, propolis and *Morus alba* gel consistently emerged as the most promising phytotherapeutic options, demonstrating the greatest magnitude of improvement across the examined periodontal indices and, in some studies, showing clinical outcomes that were comparable to or even superior to those achieved with CHX over short-term follow-up periods. In contrast to the non-selective bactericidal action of CHX, many of these products tend to support a more balanced oral microbiota by suppressing pathogenic species while preserving commensal organisms, thereby promoting a more physiological and potentially more sustainable periodontal environment. Although safety and tolerability were not primary endpoints in the included studies, several trials reported fewer or less severe adverse effects, such as tooth staining, mucosal irritation and taste disturbance, in the natural product groups than in those treated with CHX, suggesting a possibly more favorable tolerability profile that merits further investigation.

Nevertheless, because the randomized evidence available is limited to follow-up periods of up to three months, no conclusions can be drawn regarding long-term maintenance. Natural products should therefore be considered as promising adjunctive options for short-term use, while their durability and effectiveness over longer periods remain to be determined. Future research should focus on well-designed randomized controlled trials specifically investigating the most promising agents identified in this review, particularly *Morus alba*, curcumin-based formulations and propolis. The use of standardized formulations, delivery systems and longer follow-up periods will be essential to determine whether these products can be integrated into long-term periodontal maintenance protocols or considered as alternatives to CHX in selected clinical settings.

## Figures and Tables

**Table 1:** PICO framework for the systematic review comparing natural-based products and CHX as adjuncts in NSPT.

1. Participants/Population: Adolescent and adult patients diagnosed with periodontitis of any stage and grade according to the 2017 AAP/EFP classification [2].
2. Intervention/Exposure: Natural-based oral care products with antimicrobial and/or anti-inflammatory activity (e.g., herbal extracts, phytochemicals, probiotics, ozonized gels), administered as mouthwashes, gels, or toothpastes as adjuncts to NSPT.
3. Comparison/Control: CHX-based oral care products in commonly used clinical concentrations (0.12–1.0%) and formulations (mouthwashes, gels, toothpastes).
4. Outcomes: Primary outcomes, changes in periodontal clinical parameters, including PI, BoP, PPD, and CAL; Secondary outcomes: safety, tolerability, and patient-reported outcomes, when available.

**Table 2:** Inclusion and exclusion criteria followed in this review.

Inclusion Criteria	Exclusion Criteria
Study design: randomized controlled trials (parallel-group or split-mouth)	Non-primary studies (reviews, letters, editorials, case reports)
Human participants diagnosed with periodontitis (adolescents and adults, 2017 AAP/EFP classification)	In vitro or animal studies
Interventions: natural-based oral care products as adjuncts to non-surgical periodontal therapy	Studies without a comparator group using CHX
Comparator: CHX-based oral care products	Studies not reporting relevant periodontal clinical outcomes (PI, BoP, PPD, CAL) or reporting only surrogate outcomes (e.g., microbiological data without clinical measures)
Outcomes: clinical periodontal parameters (PI, BoP, PPD, CAL); secondary outcomes: safety, tolerability, patient-reported measures	Studies not evaluating periodontal therapy
No restrictions on language, clinical setting, or follow-up duration; studies published up to 30 August 2025	–

**Table 3:** Periodontal indices and scoring systems accepted in the present review.

Periodontal Parameter	Accepted Index/Measure	Scoring System/Unit	Definition/Assessment Criteria	Reference Standard
Plaque accumulation	Plaque Index (Silness & Löe)	0–3 (site-based)	Ordinal score assessing plaque thickness at the gingival margin	Silness & Löe [22]
	Plaque Control Record (PCR)	% of plaque-positive sites	Presence/absence of plaque at each site	O’Leary et al. [23]
	Turesky-modified Quigley–Hein Index	0–5 (site-based)	Ordinal assessment of plaque distribution and extent	Turesky et al. [24]
Gingival bleeding	Bleeding on Probing (BoP)	% of bleeding sites	Presence of bleeding within 10–30 s after probing	Ainamo & Bay [25]
	Sulcus Bleeding Index (SBI)	Ordinal (0–5)	Bleeding severity after gentle probing	Saxer & Mühlemann [26]
	Papillary Bleeding Index (PBI)	0–4	Bleeding intensity at interdental papilla	Saxer & Mühlemann [26]
	Gingival Bleeding Index (GBI)	Ordinal	Bleeding response to probing	Ainamo & Bay [25]
Periodontal pocket depth	Probing Pocket Depth (PPD)	Millimeters (mm)	Distance from gingival margin to pocket base	Pihlstrom [21]
Attachment level	Clinical Attachment Level (CAL)	Millimeters (mm)	Distance from CEJ or reference point to pocket base	Pihlstrom [21]

**Table 4:** Bias analysis using the ROB 2 tool [23] for randomized controlled trials.

Authors, Year of Publication, Country of Publication and Reference	D1	D2	D3	D4	D5	Overall Risk-of-Bias Judgement
Rathod et al., 2023 (India) [10]	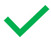	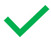	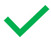	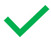	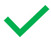	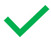
Dolly et al., 2024 (India) [11]	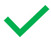	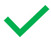	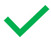	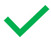	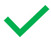	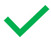
Guru et al., 2020 (India) [12]	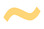	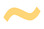	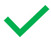	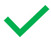	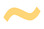	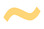
Sundaram et al., 2021 (India) [13]	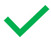	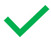	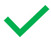	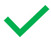	**~**	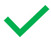
Scribante et al., 2024 (Italy) [14]	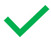	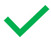	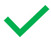	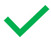	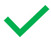	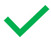
Gunjal et al., 2024 (India) [29]	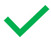	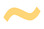	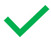	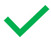	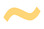	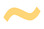
Siddharth et al., 2020 (India) [30]	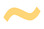	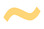	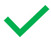	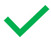	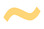	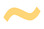
Agarwal et al., 2020 (India) [31]	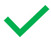	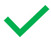	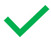	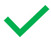	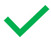	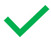
Waqar et al., 2024 (Pakistan) [32]	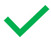	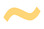	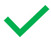	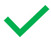	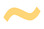	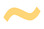
Seth et al., 2022 (India) [33]	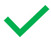	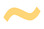	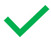	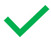	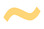	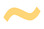
Chawla et al., 2024 (India) [34]	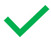	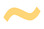	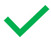	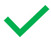	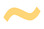	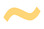

Abbreviations: D1: Bias arising from the randomization process, D2: *Bias due to deviations from intended interventions*, D3: Bias due to missing outcome data, D4: Bias in measurement of the outcome, D5: Bias in selection of the reported result, Green Symbol: Low risk of bias, Yellow Symbol: Moderate risk of bias.

**Table 5:** Clinical characteristics and outcomes of the included randomized controlled trials.

Authors, Year of Publication, Country of Publication and Reference	N° of Patients	Age (yrs)	Product	Duration	Follow-Up	CAL (mm)	PPD (mm)	BoP (%)
Rathod et al., 2023 (India) [10]	24	NR	Triphala gel	14 days	3 months	4.1 → 2.6 (*p* < 0.001) ≈ CHX	5.2 → 3.1 (*p* < 0.001) ≈ CHX	NR
Dolly et al., 2024 (India) [11]	23	30–55	Spirulina gel	14 days	3 months	3.9 → 2.5 (*p* < 0.001) ≈ CHX	5.0 → 3.2 (*p* < 0.001) ≈ CHX	NR
Guru et al., 2020 (India) [12]	45	25–50	Nanocurcumin 2% gel	21 days	45 days	4.0 → 2.4 (*p* < 0.001) ≈ CHX	5.1 → 3.0 (*p* < 0.001) ≈ CHX	42% → 18% (*p* < 0.01) ≈ CHX
Sundaram et al., 2021 (India) [13]	60	35–55	Piper betle rinse	4 weeks	3 months	4.2 → 2.7 (*p* < 0.001) ≈ CHX	5.3 → 3.3 (*p* < 0.001) ≈ CHX	48% → 22% (*p* < 0.01) ≈ CHX
Scribante et al., 2024 (Italy) [14]	30	18–70	Ozonized gel	14 days	6 months	3.8 → 2.1 (*p* < 0.001) ≈ CHX	4.9 → 2.8 (*p* < 0.001) ≈ CHX	44% → 17% (*p* < 0.001) ≈ CHX
Gunjal et al., 2024 (India) [29]	180	NR	*Morus alba* gel	21 days	3 months	4.0 → 2.2 (*p* < 0.001) ≥ CHX	5.1 → 2.9 (*p* < 0.001) > CHX	45% → 16% (*p* < 0.001) ≈ CHX
Siddharth et al., 2020 (India) [30]	25	≥30	Curcumin gel	14 days	3 months	3.9 → 2.3 (*p* < 0.001) ≥ CHX	5.0 → 3.0 (*p* < 0.001) ≈ CHX	43% → 14% (*p* < 0.001) > CHX
Agarwal et al., 2020 (India) [31]	75	30–65	Chamomile rinse	4 weeks	3 months	4.1 → 2.8 (*p* < 0.01) ≈ CHX	5.2 → 3.4 (*p* < 0.01) ≈ CHX	NR
Waqar et al., 2024 (Pakistan) [32]	144	40–50	Propolis rinse	21 days	3 months	4.0 → 2.3 (*p* < 0.001) ≈ CHX	5.0 → 3.0 (*p* < 0.001) ≈ CHX	46% → 15% (*p* < 0.001) > CHX
Seth et al., 2022 (India) [33]	20	18–55	Propolis irrigation	7 days	1 month	NR	5.2 → 3.4 (*p* < 0.01) ≈ CHX	NR
Chawla et al., 2024 (India) [34]	14	30–60	Probiotic rinse	45 days	45 days	3.8 → 2.4 (*p* < 0.01) ≈ CHX	5.0 → 3.1 (*p* < 0.01) ≈ CHX	NR
Basudan et al., 2023 (Saudi Arabia) [35]	191	NR	*Salvadora persica* rinse	6 weeks	6 weeks	NR	NR	NR
Amee et al., 2023 (India) [36]	57	NR	*Lycium barbarum* gel	21 days	3 months	4.0 → 2.6 (*p* < 0.001) ≈ CHX	5.1 → 3.2 (*p* < 0.001) ≈ CHX	NR

Abbreviations: BoP = Bleeding on Probing (% of bleeding sites); CAL = Clinical Attachment Level (mm); NR = not reported; PPD = Probing Pocket Depth (mm). Outcome ranking relative to CHX: >CHX = significant improvement compared with CHX; ≥CHX = improvement similar to or not inferior to CHX; ≈CHX = improvement comparable to CHX.

**Table 6:** Summary of study design, interventions and clinical outcomes of the included randomized controlled trials.

Authors, Year of Publication, Country of Publication and Reference	Study Design	Product	Type of Product	Propriety	Concentration	Type of Application	Clinical Outcome vs. CHX	Main Conclusion
Rathod et al. (2023), India [10]	Parallel-arm RCT	Triphala	Mouthwash	Polyherbal antioxidant and antibacterial	NR	Full-mouth disinfection adjunct	≈	Non-inferior to CHX in T2DM patients
Dolly et al. (2024), India [11]	Split-mouth RCT	Spirulina	LDD gel	Protein-rich, antioxidant, anti-inflammatory	4%	Subgingival	≈	Comparable to CHX with good safety
Guru et al. (2020), India [12]	Three-arm RCT	Curcumin nanogel	LDD nanogel	Anti-inflammatory, antioxidant, antimicrobial	2%	Periodontal pocket release	≈	Effective and safe CHX alternative
Sundaram et al. (2021), India [13]	Single-blind RCT	*Piper betle*	Mouthrinse	Antibacterial phytochemicals	2%	Post-procedural rinse	≈	Clinically comparable to CHX
Scribante et al. (2024), Italy [14]	Split-mouth RCT	Ozonized gel	O_3_-enriched lipids	Oxygen-based antimicrobial	NR	NSPT adjunct	≈	Valid CHX alternative
Gunjal et al. (2024), India [29]	Parallel-arm RCT	*Morus alba*	Extract gel	Polyphenol-rich antioxidant	16%	Subgingival	≥	Superior PPD reduction vs. CHX
Siddharth et al. (2020), India [30]	Split-mouth RCT	Curcumin	LDD gel	Anti-inflammatory and antibacterial	2%	Subgingival	≥	Equal or superior to CHX
Agarwal et al. (2020), India [31]	Parallel-arm RCT	Chamomile	Mouthwash	Flavonoid-based anti-inflammatory	1%	Post-SRP rinse	≈	Comparable with better tolerability
Waqar et al. (2024), Pakistan [32]	Double-blind RCT	Propolis	Mouthwash	Resin-based antiseptic	20%	Post-therapy rinse	>	Superior BoP reduction
Seth et al. (2022), India [33]	Parallel-arm RCT	Propolis	Subgingival irrigation	Healing and antimicrobial	25%	Subgingival	≈	Non-inferior to CHX
Chawla et al. (2024), India [34]	Double-blind RCT	Probiotics	Mouthwash	Microbiome modulation	NR	Home-care rinse	≥	Better bacterial reduction
Basudan et al. (2023), Saudi Arabia [35]	Parallel-arm RCT	*Salvadora persica*	Mouthwash	Antimicrobial plant extract	NR	Post-NSPT rinse	≈	Comparable to CHX
Amee et al. (2023), India [36]	Parallel-arm RCT	*Lycium barbarum*	Mouthwash	Antioxidant botanical	NR	Post-therapy rinse	≈	Non-inferior to CHX

Abbreviations: CHX, Chlorhexidine; LDD, Local Drug Delivery; NR, Not Reported; NSPT, Non-Surgical Periodontal Therapy; RCT, Randomized Controlled Trial; T2DM, Type 2 Diabetes Mellitus. Symbols: ≥ = similar or non-inferior; ≈ = comparable.

## Data Availability

Upon request to the corresponding author, the data are available for use.

## Supplementary Materials

The following supporting information can be downloaded at: https://www.mdpi.com/article/10.3390/dj14020110/s1, Table S1: PRISMA 2020 Checklist; Table S2: Search strategies used for each database and number of records retrieved; Table S3: Summary table of studies excluded in this systematic review; Table S4: Criteria for judging risk of bias in ROB 2 tool; Table S5: Assessment of the risk of bias specific to each domain of the ROB 2 tool; Table S6: NHLBI Quality Assessment Tool for Controlled Intervention Studies.

## Abbreviations

AAP	American Academy of Periodontology
BMI	Body Mass Index
BoP	Bleeding on probing
CAL	Clinical Attachment Level
CFU	Colony Forming Units
CHX	Chlorhexidine
EFP	European Federation of Periodontology
FBG	Fasting Blood Glucose
FMD	full mouth disinfection
GI	Gingival Index
JBI	Joanna Briggs Institute
LDD	Local Drug Delivery
MEDLINE	MEDical Literature Analysis and Retrieval System Online
MESH	Medical Subject Headings
NIH	National Institutes of Health
NHLBI	National Heart, Lung, and Blood Institute
NSAID	Non-Steroidal Anti-Inflammatory Drug
N.R.	Not Reported
NSPT	Non-Surgical Periodontal Therapy
PA	Public Administration
PCA	plaque control record
PICO	Population, Intervention, Comparison, Outcome
PI	Plaque Index
PPD	Probing Pocket Depth
PRISMA	Preferred Reporting Items for Systematic Reviews
PROSPERO	International Prospective Register of Systematic Reviews
RCT	Randomized Controlled Trial
RoB	Risk of Bias
RoB 2	Risk of Bias 2
SBI	Sulcus Bleeding Index
SRP	Scaling and Root Planing
T2DM	Type 2 Diabetes Mellitus
TIDieR	Template for Intervention Description and Replication
TVAC	Total Viable Aerobic Count
USA	United States of America
WoS	Web of Science
