# Supplementary material for: Comparison Between Natural Products and Chlorhexidine in Non-Surgical Periodontal Therapy: A Systematic Review of Randomized Clinical Trials
Source: Dent J (Basel). 2026 Feb 13;14(2):110. doi: 10.3390/dj14020110 (PMC12939140; doi:10.3390/dj14020110)
Supplement: Supplementary file 1 [file dentistry-14-00110-s001.zip › dentistry-4079130-supplementary.pdf]

Table S1. PRISMA 2020 Checklist [1].

Section and Topic	Item #	Checklist item	Location where item is reported
**TITLE**			
Title	1	Identify the report as a systematic review.	**Page 1**, Title (“*Systematic Review*”)
**ABSTRACT**			
Abstract	2	See the PRISMA 2020 for Abstracts checklist.	**Page 1**, Abstract (Objectives, Methods, Results, Conclusion clearly structured)
**INTRODUCTION**			
Rationale	3	Describe the rationale for the review in the context of existing knowledge.	**Pages 1–2**, Introduction (periodontitis burden, limits of CHX, rationale for natural products)
Objectives	4	Provide an explicit statement of the objective(s) or question(s) the review addresses.	**Page 3**, final paragraph of Introduction (explicit aim and outcomes: PI, BoP, PPD, CAL)
**METHODS**			
Eligibility criteria	5	Specify the inclusion and exclusion criteria for the review and how studies were grouped for the syntheses.	**Pages 4–5**, Section 2.4 + **Table 2**
Information sources	6	Specify all databases, registers, websites, organisations, reference lists and other sources searched or consulted to identify studies. Specify the date when each source was last searched or consulted.	**Page 3**, Section 2.3 (PubMed, Scopus, WoS; last search 30 Aug 2025)
Search strategy	7	Present the full search strategies for all databases, registers and websites, including any filters and limits used.	**Pages 3–4**, Section 2.3; full strategies in **Table S2 (Supplementary)**
Selection process	8	Specify the methods used to decide whether a study met the inclusion criteria of the review, including how many reviewers screened each record and each report retrieved, whether they worked independently, and if applicable, details of automation tools used in the process.	**Pages 4–5**, Section 2.5 (Covidence, two reviewers, consensus process)

Section and Topic	Item #	Checklist item	Location where item is reported
Data collection process	9	Specify the methods used to collect data from reports, including how many reviewers collected data from each report, whether they worked independently, any processes for obtaining or confirming data from study investigators, and if applicable, details of automation tools used in the process.	**Pages 5–6**, Section 2.6 (Excel form, independent extraction, pilot testing)
Data items	10a	List and define all outcomes for which data were sought. Specify whether all results that were compatible with each outcome domain in each study were sought (e.g. for all measures, time points, analyses), and if not, the methods used to decide which results to collect.	**Pages 3–5**, Sections 2.3–2.4 (PI, BoP, PPD, CAL; safety, tolerability)
	10b	List and define all other variables for which data were sought (e.g. participant and intervention characteristics, funding sources). Describe any assumptions made about any missing or unclear information.	**Page 6**, Section 2.6 (study characteristics, interventions, comparators)
Study risk of bias assessment	11	Specify the methods used to assess risk of bias in the included studies, including details of the tool(s) used, how many reviewers assessed each study and whether they worked independently, and if applicable, details of automation tools used in the process.	**Pages 6–7**, Section 2.7 (NHLBI + RoB 2.0, two reviewers)
Effect measures	12	Specify for each outcome the effect measure(s) (e.g. risk ratio, mean difference) used in the synthesis or presentation of results.	**Page 7**, Section 2.8 (narrative comparison of changes in clinical parameters)
Synthesis methods	13a	Describe the processes used to decide which studies were eligible for each synthesis (e.g. tabulating the study intervention characteristics and comparing against the planned groups for each synthesis (item #5)).	**Pages 4–5**, Sections 2.4–2.5
	13b	Describe any methods required to prepare the data for presentation or synthesis, such as handling of missing summary statistics, or data conversions.	**Page 7**, Section 2.8 (handling heterogeneity; no conversions/meta-analysis)
	13c	Describe any methods used to tabulate or visually display results of individual studies and syntheses.	**Pages 9–24**, Tables 3–6
	13d	Describe any methods used to synthesize results and provide a rationale for the choice(s). If meta-analysis was performed, describe the model(s), method(s) to identify the presence and extent of statistical heterogeneity, and software package(s) used.	**Pages 6–7**, Section 2.8 (narrative synthesis; rationale for no meta-analysis)
	13e	Describe any methods used to explore possible causes of heterogeneity among study results (e.g. subgroup analysis, meta-regression).	**Not performed** (explicitly stated; **Page 7**)

Section and Topic	Item #	Checklist item	Location where item is reported
	13f	Describe any sensitivity analyses conducted to assess robustness of the synthesized results.	**Not performed** (no meta-analysis; **Page 7**)
Reporting bias assessment	14	Describe any methods used to assess risk of bias due to missing results in a synthesis (arising from reporting biases).	**Pages 6–7**, Section 2.7 (RoB 2.0, selective reporting domain)
Certainty assessment	15	Describe any methods used to assess certainty (or confidence) in the body of evidence for an outcome.	**Not formally assessed** (no GRADE; limitation acknowledged **Pages 27–28**)
**RESULTS**			
Study selection	16a	Describe the results of the search and selection process, from the number of records identified in the search to the number of studies included in the review, ideally using a flow diagram.	**Pages 7–8**, Section 3 + **Figure 1 (PRISMA flow diagram)**
	16b	Cite studies that might appear to meet the inclusion criteria, but which were excluded, and explain why they were excluded.	**Page 7**, Section 3; detailed list in **Table S3 (Supplementary)**
Study characteristics	17	Cite each included study and present its characteristics.	**Pages 13–24**, Tables 4–6
Risk of bias in studies	18	Present assessments of risk of bias for each included study.	**Pages 8–12**, Section 3.1 + Tables 3, S4–S6
Results of individual studies	19	For all outcomes, present, for each study: (a) summary statistics for each group (where appropriate) and (b) an effect estimate and its precision (e.g. confidence/credible interval), ideally using structured tables or plots.	**Pages 13–21**, Tables 4 and 5
Results of syntheses	20a	For each synthesis, briefly summarise the characteristics and risk of bias among contributing studies.	**Pages 25–26**, Section 3.2
	20b	Present results of all statistical syntheses conducted. If meta-analysis was done, present for each the summary estimate and its precision (e.g. confidence/credible interval) and measures of statistical heterogeneity. If comparing groups, describe the direction of the effect.	**Pages 25–26**, Section 3.2
	20c	Present results of all investigations of possible causes of heterogeneity among study results.	**Not applicable** (no meta-analysis)
	20d	Present results of all sensitivity analyses conducted to assess the robustness of the synthesized	Not applicable

Section and Topic	Item #	Checklist item	Location where item is reported
		results.	
Reporting biases	21	Present assessments of risk of bias due to missing results (arising from reporting biases) for each synthesis assessed.	**Pages 26–27**, Discussion (publication bias discussed)
Certainty of evidence	22	Present assessments of certainty (or confidence) in the body of evidence for each outcome assessed.	**Pages 27–28**, Discussion (downgraded certainty due to heterogeneity and bias)
**DISCUSSION**			
Discussion	23a	Provide a general interpretation of the results in the context of other evidence.	**Pages 25–26**, Discussion
	23b	Discuss any limitations of the evidence included in the review.	**Pages 27–28**, Discussion
	23c	Discuss any limitations of the review processes used.	**Page 27**, Discussion
	23d	Discuss implications of the results for practice, policy, and future research.	**Pages 26–27**, Discussion
**OTHER INFORMATION**			
Registration and protocol	24a	Provide registration information for the review, including register name and registration number, or state that the review was not registered.	**Page 3**, Section 2.1 (PROSPERO CRD420251133219)
	24b	Indicate where the review protocol can be accessed, or state that a protocol was not prepared.	**Page 3**, Section 2.1
	24c	Describe and explain any amendments to information provided at registration or in the protocol.	**Not reported** (no amendments declared)
Support	25	Describe sources of financial or non-financial support for the review, and the role of the funders or sponsors in the review.	**Page 28**, Funding statement ("No external funding")
Competing interests	26	Declare any competing interests of review authors.	**Page 28**, Conflicts of Interest
Availability of data, code and other materials	27	Report which of the following are publicly available and where they can be found: template data collection forms; data extracted from included studies; data used for all analyses; analytic code; any other materials used in the review.	**Page 28**, Data Availability Statement

Table S2. Search strategies used for each database and number of records retrieved.

Database	Search Strategy	Number of Records Retrieved
PubMed (MEDLINE)	(("natural topical agents" OR "natural products" OR "plant extract*" OR "essential oils" OR phytotherapy OR herbal OR propolis OR curcumin OR aloe OR green tea OR miswak OR triphala OR grape seeds OR mangosteen OR "herbal mouthrinse" OR "plant-based therapy") AND (periodontitis OR periodontal OR "periodontal disease")) OR (("chlorhexidine" AND ("natural" OR herbal OR phytotherapy OR "plant extract*" OR alternative)) AND /periodontitis OR "periodontaltreatment")) OR (("clorhexidine" AND ("vs" OR versus OR compare*) AND ("natural product" OR phytotherapy OR "plant extract")) AND (periodontitis OR periodontal))	n = 948
Scopus	(TITLE-ABS-KEY("natural topical agents" OR "natural products" OR "plant extract*" OR "essential oils" OR phytotherapy OR herbal OR propolis OR curcumin OR aloe OR "green tea" OR miswak OR triphala OR "grape seeds" OR mangosteen OR "herbal mouthrinse" OR "plant-based therapy") AND TITLE-ABSKEY(periodontitis OR periodontal OR "periodontal disease")) OR (TITLE-ABSKEY(chlorhexidine) AND TITLE-ABSKEY("natural" OR herbal OR phytotherapy OR "plant extract*" OR alternative) AND TITLE-ABS-KEY(periodontitis OR "periodontal treatment" OR periodontal)) OR (TITLE-ABSKEY(chlorhexidine OR clorhexidine) AND TITLE-ABS-KEY(vs OR versus OR compare* OR comparison) AND TITLE-ABSKEY("natural product" OR phytotherapy OR "plant extract*" OR herbal) AND TITLEABS-KEY(periodontitis OR periodontal))	n = 1532
Web of Science (WoS)	((TS=("natural topical agents" OR "natural products" OR "plant extract*" OR "essential oils" OR phytotherapy OR herbal OR propolis OR curcumin OR aloe OR "green tea" OR miswak OR triphala OR "grape seeds" OR mangosteen OR "herbal mouthrinse" OR "plant-based therapy") AND TS=(periodontitis OR periodontal OR "periodontal disease"))) OR (TS=(chlorhexidine) AND TS=("natural" 30 OR herbal OR	n = 850

	phytotherapy OR "plant extract*" OR alternative) AND TS=(periodontitis OR "periodontal treatment" OR periodontal)) OR (TS=(chlorhexidine OR clorhexidine) AND TS=(vs OR versus OR compare* OR comparison) AND TS=("natural product" OR phytotherapy OR "plant extract*" OR herbal) AND TS=(periodontitis OR periodontal))	

Table S3. Summary table of studies excluded in this systematic review.

Excluded Studies	Exclusion Reasons
Beyer et al., 2020 [2]	Intervention not relevant to the research question
Junger et al., 2020 [3]	Population different from that with periodontitis
Borca et al., 2024 [4]	Intervention not relevant to the research question
Farjana et al., 2025 [5]	Intervention not relevant to the research question
Phumat et al., 2022 [6]	Population different from that with periodontitis
Mathew et al., 2023 [7]	Studies on synthetic or non-dental drugs
Soulissa et al., 2021 [8]	Absence of comparison with chlorhexidine
Kharaeva et al., 2020 [9]	Population different from that with periodontitis
Heuzeroth et al., 2025 [10]	Intervention not relevant to the research question
Guenther et al., 2022 [11]	Intervention not relevant to the research question
Wang et al., 2021 [12]	Absence of comparison with chlorhexidine
Sha et al., 2021 [13]	Intervention not relevant to the research question
Prasanna et al., 2025 [14]	Intervention not relevant to the research question
McCullough et al., 2023 [15]	Intervention not relevant to the research question
Sultana et al., 2024 [16]	Intervention not relevant to the research question
Choi et al., 2020 [17]	Studies on synthetic or non-dental drugs
Nugraha et al., 2023 [18]	Intervention not relevant to the research question
Kumbar et al., 2021 [19]	Intervention not relevant to the research question
Shaheen et al., 2023 [20]	Absence of comparison with chlorhexidine
Izui et al., 2021 [21]	Intervention not relevant to the research question
Barros et al., 2020 [22]	Studies on non-relevant dental procedures
Ramesh et al., 2024 [23]	Studies on non-relevant dental procedures
Rezvani et al., 2022 [24]	Studies on non-relevant dental procedures
George et al., 2021 [25]	Absence of comparison with chlorhexidine
Tennert et al., 2020 [26]	Absence of comparison with chlorhexidine
Sidharta et al., 2024 [27]	Studies on non-relevant dental procedures
Boyapati et al., 2024 [28]	Population different from that with periodontitis
Al-Zawawi et al., 2022 [29]	Intervention not relevant to the research question
Shi et al., 2021 [30]	Studies on non-relevant dental procedures

Kameri et al., 2024 [31]	Studies on non-relevant dental procedures
Abdallah et al., 2024 [32]	Intervention not relevant to the research question
Duane et al., 2023 [33]	Intervention not relevant to the research question
Takada et al., 2024 [34]	Intervention not relevant to the research question
Qamara et al., 2021 [35]	Intervention not relevant to the research question
Chansamart et al., 2023 [36]	Intervention not relevant to the research question
Maximo et al., 2020 [37]	Intervention not relevant to the research question
Wilder et al., 2022 [38]	Intervention not relevant to the research question
Guzman-Florence et al., 2023 [39]	Studies on synthetic or non-dental drugs
Perez-Pacheco et al., 2021 [40]	Studies on non-relevant dental procedures
Rath et al., 2024 [41]	Studies on synthetic or non-dental drugs
Maybodi et al., 2025 [42]	Intervention not relevant to the research question
Abullais et al., 2022 [43]	Intervention not relevant to the research question
Stańdo-Retecka et al., 2023 [44]	Absence of comparison with chlorhexidine
Yao et al., 2021 [45]	Studies on non-relevant dental procedures
Cankaya et al., 2025 [46]	Studies on non-relevant dental procedures
Manjunatha et al., 2022 [47]	Intervention not relevant to the research question
Assiry et al., 2021 [48]	Intervention not relevant to the research question
Kaplan et al., 2021 [49]	Studies on non-relevant dental procedures
Kim et al., 2022 [50]	Intervention not relevant to the research question
De Rossi et al., 2021 [51]	Studies on non-relevant dental procedures
Boyapati et al., 2024 [52]	Population different from that with periodontitis
Pardiñas et al., 2025 [53]	Intervention not relevant to the research question
Al-Askar et al., 2022 [54]	Intervention not relevant to the research question
Park et al., 2021 [55]	Population different from that with periodontitis
Andhare et al., 2024 [56]	Population different from that with periodontitis
Zuttion et al., 2024 [57]	Population different from that with periodontitis
Wasti et al., 2021 [58]	Intervention not relevant to the research question
Jung et al., 2024 [59]	Population different from that with periodontitis
Bayer et al., 2025 [60]	Intervention not relevant to the research question
Kim et al., 2025 [61]	Studies on synthetic or non-dental drugs

Corbella et al., 2024 [62]	Population different from that with periodontitis
Pérez-Pacheco et al., 2024 [63]	Absence of comparison with chlorhexidine
Bakhshi et al., 2022 [64]	Studies on non-relevant dental procedures
Al-Mahmood et al., 2021 [65]	Population different from that with periodontitis
Senkalvarayan et al., 2023 [66]	Population different from that with periodontitis
Milleman et al., 2022 [67]	Intervention not relevant to the research question
Srikumar et al., 2022 [68]	Intervention not relevant to the research question
Erbasar et al., 2023 [69]	Studies on non-relevant dental procedures
Al-Zawawi et al., 2022 [70]	Intervention not relevant to the research question
Kamath et al., 2023 [71]	Population different from that with periodontitis
Alqutub et al., 2023 [72]	Population different from that with periodontitis
Ahu et al., 2023 [73]	Absence of comparison with chlorhexidine
Amano et al., 2025 [74]	Absence of comparison with chlorhexidine
Abdel-Fatah et al., 2023 [75]	Absence of comparison with chlorhexidine
Sutthiboonyapan et al., 2025 [76]	Population different from that with periodontitis
Eltantawi et al., 2024 [77]	Studies on non-relevant dental procedures
Kiani et al., 2022 [78]	Population different from that with periodontitis
Valkenburg et al., 2021 [79]	Population different from that with periodontitis
Qamar et al., 2021 [80]	Absence of comparison with chlorhexidine
Katanasaka et al., 2021 [81]	Absence of comparison with chlorhexidine
Khabazian et al., 2025 [82]	Population different from that with periodontitis
Bapat et al., 2023 [83]	Studies on synthetic or non-dental drugs
Abdul-Wahab et al., 2025 [84]	Population different from that with periodontitis
González-Serrano et al., 2021 [85]	Population different from that with periodontitis
Cosola et al., 2022 [86]	Population different from that with periodontitis
Kim et al., 2025 [87]	Population different from that with periodontitis
Taalab et al., 2021 [88]	Absence of comparison with chlorhexidine
Azizan et al., 2023 [89]	Population different from that with periodontitis
Chaganti et al., 2023 [90]	Studies on non-relevant dental procedures
Bolla et al., 2023 [91]	Intervention not relevant to the research question
Oliva et al., 2023 [92]	Population different from that with periodontitis

Hussein et al., 2022 [93]	Studies on synthetic or non-dental drugs
Derman et al., 2021 [94]	Studies on synthetic or non-dental drugs
Sayar et al., 2021 [95]	Population different from that with periodontitis
Eltay et al., 2021 [96]	Population different from that with periodontitis
Vitiello et al., 2024 [97]	Intervention not relevant to the research question
Potewiratnanond et al., 2025 [98]	Studies on non-relevant dental procedures
Laky et al., 2024 [99]	Absence of comparison with chlorhexidine
Stańdo-Retecka et al., 2023 [100]	Absence of comparison with chlorhexidine
Matos et al., 2024 [101]	Population different from that with periodontitis
Fan et al., 2023 [102]	Studies on non-relevant dental procedures
Yaghmoor et al., 2024 [103]	Studies on non-relevant dental procedures
Fahim et al., 2024 [104]	Absence of comparison with chlorhexidine
Weber et al., 2024 [105]	Population different from that with periodontitis
Mathew et al., 2023 [106]	Absence of comparison with chlorhexidine
Nandlal et al., 2021 [107]	Absence of comparison with chlorhexidine
Jockel-Schneider et al., 2024 [108]	Population different from that with periodontitis
Birjandi-Anahid et al., 2025 [109]	Intervention not relevant to the research question
Deesricharoenkiat et al., 2022 [110]	Studies on non-relevant dental procedures
Zhang et al., 2023 [111]	Studies on non-relevant dental procedures
Chaubal et al., 2024 [112]	Absence of comparison with chlorhexidine
Abdallah-Khalil et al., 2024 [113]	Intervention not relevant to the research question
Zandesh et al., 2025 [114]	Studies on non-relevant dental procedures
Baltieri et al., 2024 [115]	Studies on non-relevant dental procedures
Talasani et al., 2022 [116]	Population different from that with periodontitis
Kose et al., 2025 [117]	Population different from that with periodontitis
Kamath et al., 2023 [118]	Studies on non-relevant dental procedures
Huang et al., 2022 [119]	Absence of comparison with chlorhexidine
Almutairi et al., 2022 [120]	Intervention not relevant to the research question
Cavagni et al., 2021 [121]	Population different from that with periodontitis
Lorusso et al., 2022 [122]	Studies on synthetic or non-dental drugs
Hermes et al., 2024 [123]	Studies on synthetic or non-dental drugs

Sharma et al., 2021 [124]	Absence of comparison with chlorhexidine
Ebrookes et al., 2023 [125]	Intervention not relevant to the research question
Moradi et al., 2023 [126]	Studies on synthetic or non-dental drugs
Al-Abdullah et al., 2022 [127]	Studies on non-relevant dental procedures
Hameeda et al., 2020 [128]	Studies on non-relevant dental procedures
Souza et al., 2020 [129]	Studies on non-relevant dental procedures
Lan et al., 2023 [130]	Studies on non-relevant dental procedures
Nam et al., 2023 [131]	Studies on non-relevant dental procedures
Marconcini et al., 2023 [132]	Studies on synthetic or non-dental drugs
Saini et al., 2025 [133]	Absence of comparison with chlorhexidine
Stanton et al., 2021 [134]	Studies on synthetic or non-dental drugs
Dai et al., 2025 [135]	Studies on non-relevant dental procedures
Bello et al., 2020 [136]	Population different from that with periodontitis
Noushad et al., 2020 [137]	Absence of comparison with chlorhexidine
Damayanti et al., 2020 [138]	Studies on non-relevant dental procedures
Ji et al., 2021 [139]	Studies on non-relevant dental procedures
Aherne et al., 2022 [140]	Studies on synthetic or non-dental drugs
Rawat et al., 2024 [141]	Population different from that with periodontitis
Rodrigues et al., 2025 [142]	Studies on non-relevant dental procedures
Herdiyati et al., 2020 [143]	Studies on non-relevant dental procedures
Bunwanna et al., 2021 [144]	Studies on non-relevant dental procedures
Kommuri et al., 2022 [145]	Population different from that with periodontitis
Rodrigues et al., 2023 [146]	Studies on non-relevant dental procedures
Deesricharoenkiat et al., 2022 [147]	Studies on non-relevant dental procedures
Cheng et al., 2022 [148]	Studies on non-relevant dental procedures
Tanuguchi et al., 2025 [149]	Population different from that with periodontitis
Eltantawi et al., 2024 [150]	Studies on non-relevant dental procedures
Garcia et al., 2022 [151]	Studies on non-relevant dental procedures
Ghavimi et al., 2020 [152]	Studies on non-relevant dental procedures
Zhang et al., 2023 [153]	Absence of comparison with chlorhexidine
Abullais Saquib et al., 2021 [154]	Studies on synthetic or non-dental drugs

Strappa et al., 2022 [155]	Studies on non-relevant dental procedures
Grant et al., 2023 [156]	Studies on non-relevant dental procedures
Calabrese et al., 2021 [157]	Studies on non-relevant dental procedures
Scott et al., 2022 [158]	Population different from that with periodontitis
Zuttion et al., 2024 [159]	Population different from that with periodontitis
Vo et al., 2021 [160]	Studies on non-relevant dental procedures
Yoshida et al., 2022 [161]	Intervention not relevant to the research question
Yagci et al., 2021 [162]	Studies on non-relevant dental procedures
Alonso-Español et al., 2025 [163]	Studies on non-relevant dental procedures
Marya et al., 2022 [164]	Absence of comparison with chlorhexidine
Dinesh et al., 2024 [165]	Studies on synthetic or non-dental drugs
Fakhri et al., 2023 [166]	Studies on non-relevant dental procedures
Moghadam et al., 2022 [167]	Studies on non-relevant dental procedures
Delavarian et al., 2023 [168]	Studies on non-relevant dental procedures
Alipour et al., 2021 [169]	Studies on non-relevant dental procedures
Tambur et al., 2021 [170]	Population different from that with periodontitis
Kumar et al., 2022 [171]	Population different from that with periodontitis
Atila et al., 2024 [172]	Studies on non-relevant dental procedures
De Araújo et al., 2024 [173]	Studies on non-relevant dental procedures
Luo et al., 2025 [174]	Studies on synthetic or non-dental drugs
Justo et al., 2022 [175]	Studies on non-relevant dental procedures
Derman et al., 2021 [176]	Population different from that with periodontitis
Moradi et al., 2023 [177]	Studies on synthetic or non-dental drugs
Reis et al., 2020 [178]	Studies on non-relevant dental procedures
Cirano et al., 2021 [179]	Studies on synthetic or non-dental drugs
Kendell-Wall et al., 2024 [180]	Studies on non-relevant dental procedures
Neto et al., 2021 [181]	Studies on synthetic or non-dental drugs
Khalil et al., 2025 [182]	Studies on non-relevant dental procedures
Zheng et al., 2024 [183]	Studies on non-relevant dental procedures
Nittayananta et al., 2023 [184]	Absence of comparison with chlorhexidine
Prado et al., 2022 [185]	Population different from that with periodontitis

Yao et al., 2021 [186]	Studies on synthetic or non-dental drugs
BinShabaib et al., 2022 [187]	Absence of comparison with chlorhexidine
Blank et al., 2021 [188]	Population different from that with periodontitis

Table S4. Criteria for judging risk of bias in ROB 2 tool [189].

Section/Domain	Description	Key Questions / Criteria	Response Options / Risk of Bias Judgement
Domain 1: Randomization process	Risk of bias arising from the randomization process.	**1.1** Was the allocation sequence random? **1.2** Was the allocation sequence concealed until participants were enrolled and assigned to interventions? **1.3** Did baseline differences between intervention groups suggest a problem with the randomization process?	**Responses:** Y / PY / PN / N / NI • **Judgement:** Low / Some concerns / High
Domain 2 a(effect of assignment): Deviations from intended interventions	Risk of bias due to deviations from intended interventions when estimating the **effect of assignment** (intention-to-treat).	**2.1** Were participants aware of their assigned intervention during the trial? **2.2** Were carers/people delivering the interventions aware of participants' assigned intervention? **2.3** *(if Y/PY/NI to 2.1 or 2.2)* Were there deviations from intended interventions arising because of the trial context? **2.4** *(if Y/PY to 2.3)* Were these deviations likely to have affected the outcome? **2.5** *(if Y/PY/NI to 2.4)* Were these deviations balanced between groups? **2.6** Was an appropriate analysis used to estimate the **effect of assignment**? **2.7** *(if N/PN/NI to 2.6)* Was there potential for a substantial impact on the result due to failure to analyse participants in their randomized groups?	**Responses:** Y / PY / PN / N / NI (NA where indicated) • **Judgement:** Low / Some concerns / High
Domain 2b (effect of adherence): Deviations from intended interventions	Risk of bias due to deviations from intended interventions when estimating the **effect of adhering to intervention** (per-protocol).	**2.1** Were participants aware of their assigned intervention during the trial? **2.2** Were carers/people delivering the interventions aware of participants' assigned intervention? **2.3 (if applicable)** Were important non-protocol interventions balanced across groups? **2.4 (if applicable)** Were there failures in implementing the intervention that could have affected the outcome? **2.5 (if applicable)** Was	**Responses:** Y / PY / PN / N / NI (NA where indicated) • **Judgement:** Low / Some concerns / High

		there non-adherence to the assigned intervention regimen that could have affected outcomes? **2.6 (if N/PN/NI to 2.3, or Y/PY/NI to 2.4/2.5)** Was an appropriate analysis used to estimate the **effect of adherence**?	
Domain 3: Missing outcome data	Risk of bias due to missing outcome data.	**3.1** Were data for this outcome available for all, or nearly all, randomized participants? **3.2 (if N/PN/NI to 3.1)** Is there evidence that the result was **not** biased by missing outcome data? **3.3 (if N/PN to 3.2)** Could missingness in the outcome depend on its true value? **3.4 (if Y/PY/NI to 3.3)** Is it **likely** that missingness in the outcome depended on its true value?	**Responses:** Y / PY / PN / N / NI (NA where indicated) • **Judgement:** Low / Some concerns / High
Domain 4: Measurement of the outcome	Risk of bias in measurement of the outcome.	**4.1** Was the method of measuring the outcome inappropriate? **4.2** Could measurement or ascertainment of the outcome have differed between groups? **4.3 (if N/PN/NI to 4.1 and 4.2)** Were outcome assessors aware of the intervention received by participants? **4.4 (if Y/PY/NI to 4.3)** Could assessment of the outcome have been influenced by knowledge of the intervention received? **4.5 (if Y/PY/NI to 4.4)** Is it **likely** that assessment of the outcome was influenced by knowledge of the intervention received?	**Responses:** Y / PY / PN / N / NI (NA where indicated) • **Judgement:** Low / Some concerns / High
Domain 5: Selection of the reported result	Risk of bias in selection of the reported result.	**5.1** Were the data that produced this result analysed in accordance with a **pre-specified analysis plan** finalized before unblinded outcome data were available? **5.2** Is the numerical result likely to have been selected, based on the results, from **multiple eligible outcome measurements** within the domain? **5.3** Is the numerical result likely to have been selected, based on the results, from **multiple eligible analyses** of the data?	**Responses:** Y / PY / PN / N / NI • **Judgement:** Low / Some concerns / High

Overall Risk of Bias	Overall judgement derived from all domains.	--	**Rules:** Low = all domains Low • Some concerns = ≥1 domain Some concerns and **no** High • High = ≥1 domain High

Table S5. Assessment of the risk of bias specific to each domain of the ROB 2 tool [189].

Reference (first author et all., year)	D1 (1.1/1.2/1.3)	D2 (Assignment) (2.1–2.7)	D2 (Adherence) (2.1–2.6)	D3 (3.1/3.2/3.3/3.4)	D4 (4.1/4.2/4.3/4.4/4.5)	D5 (5.1/5.2/5.3)
Gunjal et all., 2024 [190]	PY/NI/N	PY/PN/PN/NI/NA/NA/NA	NA/NA/NA/NA/NA/NA	PY/PY/PY/NA	Y/PY/PN/NA/NA	NI/NI/PN
Sundaram et all., 2021 [191]	PY/NI/N	NI/PN/PN/NI/NA/NA/NA	NA/NA/NA/NA/NA/NA	PY/PY/PY/NA	Y/NI/PN/NA/NA	NI/NI/PN
Siddharth et all., 2020 [192]	PY/NI/N	NI/PN/PN/NI/NA/NA/NA	NA/NA/NA/NA/NA/NA	PY/PY/PY/NA	Y/NI/PN/NA/NA	NI/NI/PN
Agarwal et all., 2020 [193]	Y/PY/N	PY/PN/PN/NI/NA/NA/NA	NA/NA/NA/NA/NA/NA	PY/PY/PY/NA	Y/PY/PN/NA/NA	NI/NI/PN
Dolly et all., 2024 [194]	PY/NI/N	PY/PN/PN/NI/NA/NA/NA	NA/NA/NA/NA/NA/NA	PY/PY/PY/NA	Y/NI/PN/NA/NA	NI/NI/PN
Scribante et all., 2024 [195]	Y/PY/N	PY/PN/PN/PY/NA/NA/NA	NA/NA/NA/NA/NA/NA	PY/PY/PY/NA	Y/PY/PN/NA/NA	NI/NI/PN
Waqar et all., 2024 [196]	PY/PY/N	PY/PN/PN/PY/NA/NA/NA	NA/NA/NA/NA/NA/NA	PY/PY/PY/NA	Y/PY/PN/NA/NA	NI/NI/PN
Seth et all., 2022 [197]	PY/NI/N	PY/PN/PN/NI/NA/NA/NA	NA/NA/NA/NA/NA/NA	PY/PY/PY/NA	Y/PY/PN/NA/NA	NI/NI/PN

Guru et all., 2020 [198]	PY/NI/N	NI/PN/PN/NI/NA/NA/NA	NA/NA/NA/NA/NA/NA	PY/PY/PY/NA	Y/NI/PN/NA/NA	NI/NI/PN
Rathod et all., 2023 [199]	PY/NI/N	PY/PN/PN/PY/NA/NA/NA	NA/NA/NA/NA/NA/NA	PY/PY/PY/NA	Y/PY/PN/NA/NA	NI/NI/PN
Chawla et all., 2024 [200]	Y/PY/N	PY/PN/PN/PY/NA/NA/NA	NA/NA/NA/NA/NA/NA	PY/PY/PY/NA	Y/PY/PN/NA/NA	NI/NI/PN
Basudan et all., 2023 [201]	PY/PY/N	PY/PN/PN/NI/NA/NA/NA	NA/NA/NA/NA/NA/NA	PY/PY/PY/NA	Y/PY/PN/NA/NA	NI/NI/PN
Amee et all., 2024 [202]	PY/NI/N	PY/PN/PN/NI/NA/NA/NA	NA/NA/NA/NA/NA/NA	PY/PY/PY/NA	Y/PY/PN/NA/NA	NI/NI/PN

Abbreviations: D1: Bias due to the randomization process; D2-A = Effect of assignment (intention-to-treat), D2-B = Effect of adherence (per-protocol). D3: Bias due to missing outcome data; D4 Bias in measurement of the outcome; D5 = Bias in selection of the reported result.

Table S6. NHLBI Quality Assessment Tool for Controlled Intervention Studies [203].

First Author et al., Year	Q1	Q2	Q3	Q4	Q5	Q6	Q7	Q8	Q9	Q10	Q11	Q12	Q13	Q14	Total Score	Quality Rating
Gunjal, 2024 [190]	Y	Y	Y	Y	Y	Y	Y	Y	Y	Y	Y	N	N	N	11/12	Good
Sundaram 2021 [191]	Y	Y	Y	Y	Y	Y	Y	Y	Y	N	N	N	N	N	9/12	Fair
Siddharth 2020 [192]	Y	Y	Y	Y	Y	Y	Y	Y	Y	Y	N	N	N	N	10/12	Fair
Agarwal 2020 [193]	Y	Y	Y	Y	Y	Y	Y	Y	N	N	N	N	N	N	8/12	Fair
Dolly 2024 [194]	Y	Y	Y	Y	Y	Y	Y	Y	Y	N	N	N	N	N	9/12	Fair
Scribante 2024 [195]	Y	Y	Y	Y	Y	Y	Y	Y	Y	Y	Y	Y	N	N	12/1	Good
Waqar 2024 [196]	Y	Y	Y	Y	Y	Y	Y	Y	Y	Y	Y	N	N	N	11/12	Good
Seth 2022 [197]	Y	Y	Y	Y	Y	Y	Y	Y	N	N	N	N	N	N	8/12	Fair
Guru	Y	Y	Y	Y	Y	Y	Y	Y	Y	Y	N	N	N	N	10/12	Fair

2020 [198]																
Rathod 2023 [199]	Y	Y	Y	Y	Y	Y	Y	Y	Y	N	N	N	N	N	9/12	Fair
Chawla 2024 [200]	Y	Y	Y	Y	Y	Y	Y	N	N	N	N	N	N	N	7/12	Fair
Basudan 2023 [201]	Y	Y	Y	Y	Y	Y	Y	Y	N	N	N	N	N	N	8/12	Fair
Amee 2023 [202]	Y	Y	Y	Y	Y	Y	Y	Y	Y	Y	N	N	N	N	10/12	Fair

Q1: Was the study's objective or research question clearly stated and appropriate?, Q2: Was the study population clearly specified and defined (with inclusion and exclusion criteria adequately reported)?, Q3: Was the participation rate of eligible subjects sufficiently high (adequate participation or follow-up rate)?, Q4: Were the study participants selected in a manner that made them representative of the target population?, Q5: Was the sample size justified or adequate to address the research question?, Q6: Were the exposures or interventions of interest clearly defined, valid, reliable, and consistently applied to all participants?, Q7: Were the outcomes of interest clearly defined, valid, reliable, and consistently measured across all participants?, Q8: Was the length of follow-up sufficient to observe the effects of the intervention or exposure?, Q9: Were losses to follow-up adequately reported and analyzed?, Q10: Were outcome assessors blinded to the exposure status or group assignment of participants?, Q11: Were key potential confounding variables identified, measured, and statistically controlled for in the analysis (or adequately managed through matching)?, Q12: Were the statistical analyses appropriate for the study design and the type of data collected?, Q13: Were results clearly and comprehensively reported for all variables and study groups?, Q14: Were the study conclusions justified by the results obtained and supported by the data presented? Total Score: Number of yes; N: no; Y: yes. Quality Rating: Poor <50%, Fair 50–75%, Good ≥75%.

References

1. Page MJ, McKenzie JE, Bossuyt PM, Boutron I, Hoffmann TC, Mulrow CD, Shamseer L, Tetzlaff JM, Akl EA, Brennan SE, Chou R, Glanville J, Grimshaw JM, Hróbjartsson A, Lalu MM, Li T, Loder EW, Mayo-Wilson E, McDonald S, McGuinness LA, Stewart LA, Thomas J, Tricco AC, Welch VA, Whiting P, Moher D. The PRISMA 2020 statement: an updated guideline for reporting systematic reviews. BMJ 2021; 372: n71. doi: 10.1136/bmj. n7.
2. Beyer, A., Dalton, M., Doll, K., Winkel, A., Stumpp, N. S., & Stiesch, M. (2020). In Vitro Antibacterial Effectiveness of a Naturopathic Oral Care Product on Oral Pathogens. Oral health & preventive dentistry, 18(3), 625–632. https://doi.org/10.3290/j.ohpd.a44938
3. Jünger, H., Jaun-Ventrice, A., Guldener, K., Ramseier, C. A., Reissmann, D. R., & Schimmel, M. (2020). Anti-inflammatory potential of an essential oil-containing mouthwash in elderly subjects enrolled in supportive periodontal therapy: a 6-week randomised controlled clinical trial. Clinical oral investigations, 24(9), 3203–3211. https://doi.org/10.1007/s00784-019-03194-3
4. Borcă, M., Borcă, A., Romila, L., Ciobica, A., Mavroudis, I., Tomida, M., ... & Forna, N. (2024). ANTIOXIDANT EFFECTS OF MEDICINAL PLANTS WITH APPLICABILITY IN ORO-DENTAL MEDICINE. Romanian Journal of Oral Rehabilitation, 16(2).
5. H. Nilofer Farjana, G. Mohan Valiathan, S. Mohanasatheesh,In-silico study on biomolecules derived from Cissus quadrangularis towards anti-inflammation,Journal of Genetic Engineering and Biotechnology,Volume 23, Issue 4,2025,100571, ISSN 1687-157X, https://doi.org/10.1016/j.jgeb.2025.100571.
6. Phumat, P., Khongkhunthian, S., Wanachantararak, P., & Okonogi, S. (2020). Comparative inhibitory effects of 4-allylpyrocatechol isolated from Piper betle on Streptococcus intermedius, Streptococcus mutans, and Candida albicans. Archives of oral biology, 113, 104690. https://doi.org/10.1016/j.archoralbio.2020.104690
7. Mathew, C. A., Veena, H. R., Shubha, P., & Daniel, R. A. (2023). Antimicrobial photocatalysis using bio-hydrothermally synthesized Zinc oxide nanoparticles in the management of periodontitis: a prospective split-mouth, double-blind, randomized, controlled clinical trial. Journal of applied oral science : revista FOB, 31, e20230271. https://doi.org/10.1590/1678-7757-2023-0271
8. Soulissa, AG, Lombardo, B., & Widyarman, A. Efficacia antibatterica e antibiofilm della gobba di ananas (Ananas comosus) su Porphyromonas gingivalis in vitro. J Dent Indones. 2021;28(3): 153-157
9. Kharaeva, ZF, Mustafaev, MS, Khazhmetov, AV, Gazaev, IH, Blieva, LZ, Steiner, L., Mayer, W., De Luca, C. e Korkina, LG (2020). Effetti antibatterici e antinfiammatori del dentifricio con erbe medicinali svizzere nei pazienti affetti da gengivite e fase iniziale di parodontite: dall'efficacia clinica ai meccanismi. Dentistry Journal , 8 (1), 10. https://doi.org/10.3390/dj8010010
10. Heuzeroth, G., Kaufmann, ME, Herter-Aeberli, I. et al. Valutazione del potenziale dei composti "superfood" del tè verde o della curcuma come coadiuvanti rispetto agli approcci terapeutici consolidati per la malattia parodontale. Clin Oral Invest 29 , 61 (2025). https://doi.org/10.1007/s00784-024-06122-2
11. Günther, M., Karygianni, L., Argyropoulou, A., Anderson, A. C., Hellwig, E., Skaltsounis, A. L., Wittmer, A., Vach, K., & Al-Ahmad, A. (2022). The antimicrobial effect of Rosmarinus officinalis extracts on oral initial adhesion ex vivo. Clinical oral investigations, 26(6), 4369–4380. https://doi.org/10.1007/s00784-022-04400-5
12. Wang, Y., Zeng, J., Yuan, Q., & Luan, Q. (2021). Efficacy of (-)-epigallocatechin gallate delivered by a new-type scaler tip during scaling and root planing on chronic periodontitis: a split-mouth, randomized clinical trial. BMC oral health, 21(1), 79. https://doi.org/10.1186/s12903-021-01418-1
13. Sha, A. M., Garib, B. T., Azeez, S. H., & Gul, S. S. (2021). Effects of curcumin gel on osteoclastogenic bone markers in experimental periodontitis and alveolar bone loss in wistar rats. Journal of dental sciences, 16(3), 905–914. https://doi.org/10.1016/j.jds.2020.09.015

14. Prasanna, Jammula Surya; SaiPriya, Jilkapally .Valutazione dell'efficacia dei chip a base di erbe (neem) e di sostanze chimiche (clorexidina) come agenti di somministrazione locale di farmaci nel trattamento della parodontite cronica: uno studio clinico e microbiologico. Journal of Oral Research and Review 17(1):p 35-46, gennaio-giugno 2025. | DOI: 10.4103/jorr.jorr_46_24
15. Brookes, Z. L. S., McCullough, M., Kumar, P., & McGrath, C. (2023). Mouthwashes: Implications for Practice. International dental journal, 73 Suppl 2(Suppl 2), S98–S101. https://doi.org/10.1016/j.identj.2023.08.013
16. Sultana, Amra; Tasneem, Anam; Krishnappa, Pushpanjali; Shwetha, KM .Efficacia del collutorio a base di Moringa oleifera nei giovani adulti come agente antiplacca: uno studio interventistico. Rivista dell'Associazione indiana di odontoiatria sanitaria pubblica 22(2):p 185-190, aprile-giugno 2024. | DOI: 10.4103/jiaphd.jiaphd_242_23
17. Choi, G. E., & Hyun, K. Y. (2020). Inhibitory effect of Acer tegmentosum maxim extracts on P. gingivalis LPS-induced periodontitis. Archives of oral biology, 109, 104529. https://doi.org/10.1016/j.archoralbio.2019.104529
18. Nugraha, A. P., Sibero, M. T., Nugraha, A. P., Puspitaningrum, M. S., Rizqianti, Y., Rahmadhani, D., Kharisma, V. D., Ramadhani, N. F., Ridwan, R. D., Noor, T. N. E. B. T. A., & Ernawati, D. S. (2023). Anti-Periodontopathogenic Ability of Mangrove Leaves (Aegiceras corniculatum) Ethanol Extract: In silico and in vitro study. European journal of dentistry, 17(1), 46–56. https://doi.org/10.1055/s-0041-1741374
19. Kumbar, V. M., Peram, M. R., Kugaji, M. S., Shah, T., Patil, S. P., Muddapur, U. M., & Bhat, K. G. (2021). Effect of curcumin on growth, biofilm formation and virulence factor gene expression of Porphyromonas gingivalis. Odontology, 109(1), 18–28. https://doi.org/10.1007/s10266-020-00514-y
20. Shaheen, M. Y., Al-Zawawi, A. S., Divakar, D. D., Aldulaijan, H. A., & Basudan, A. M. (2023). Role of Chlorhexidine and Herbal Oral Rinses in Managing Periodontitis. International dental journal, 73(2), 235–242. https://doi.org/10.1016/j.identj.2022.06.027
21. Izui, S., Sekine, S., Murai, H., Takeuchi, H., & Amano, A. (2021). Inhibitory effects of curcumin against cytotoxicity of Porphyromonas gingivalis outer membrane vesicles. Archives of oral biology, 124, 105058. https://doi.org/10.1016/j.archoralbio.2021.105058
22. Barros, S. P., Hefni, E., Fahimipour, F., Kim, S., & Arora, P. (2020). Maintaining barrier function of infected gingival epithelial cells by inhibition of DNA methylation. Journal of periodontology, 91 Suppl 1, S68–S78. https://doi.org/10.1002/JPER.20-0262
23. Ramesh, K., Joshna, I., Penmetsa, GS, Sruthima, GN, Kumar, PM, Anusha, B. e Valli, S. Estratto di buccia di mangostano vs gel di tetraciclina nel trattamento dell'ascesso parodontale nel diabete di tipo 2: uno studio clinico randomizzato. J Dent Indones. 2024;31(1): 17-25
24. Rezvani, G., Taleghani, F., & Valizadeh, M. (2022). Effect of Green Tea on the Level of Salivary Interleukin-1 Beta in Patients with Chronic Periodontitis: A Randomized Clinical Trial. International journal of dentistry, 2022, 8992313. https://doi.org/10.1155/2022/8992313
25. George, Priyanka Mariam; Jayakumar, Nadathur Doraisamy; Kaarthikeyan, Gurumoorthy .Efficacia delle nanofibre elettrofilate di Ocimum Sanctum come coadiuvante della detartrasi e della levigatura radicolare nella gestione della parodontite cronica: Uno studio clinico randomizzato controllato. Rivista di salute orale internazionale 13(2):p 115-121, marzo-aprile 2021. | DOI: 10.4103/jioh.jioh_245_2
26. Woelber, J. P., & Tennert, C. (2020). Chapter 13: Diet and Periodontal Diseases. Monographs in oral science, 28, 125–133. https://doi.org/10.1159/000455380
27. Rani Safitri, C., Sidharta, W., Kusumaningsih, A., Wicaksono, F. M., Aljunaid, M., & Krismariono, A. (2024). The effect of curcumin 1% methanolic extract on the expression of Matrix Metalloproteinase-1, Matrix Metalloproteinase-8, Matrix Metalloproteinase-13, neutrophil, macrophage, lymphocyte counts in Porphyromonas gingivalis induced periodontitis: a randomized controlled trial. The Saudi dental journal, 36(10), 1339–1343. https://doi.org/10.1016/j.sdentj.2024.08.002

28. Boyapati, R., Peeta, J., Dhulipalla, R., Kolaparthy, L., Adurty, C., & Cheruvu, R. N. S. (2024). Comparative evaluation of the efficacy of probiotic, Aloe vera, povidine-iodine, and chlorhexidine mouthwashes in the treatment of gingival inflammation: A randomized controlled trial. Dental and medical problems, 61(2), 181–190. https://doi.org/10.17219/dmp/156425
29. Al-Zawawi, A. S., Shaheen, M. Y., Divakar, D. D., Aldulaijan, H. A., & Basudan, A. M. (2022). Postoperative anti-inflammatory efficacy of 2% saline rinses and a herbal- mouthwash after non-surgical periodontal therapy for the management of periodontal inflammation in young adults with chlorhexidine allergy: A randomized controlled trial. International journal of dental hygiene, 20(2), 408–414. https://doi.org/10.1111/idh.12583
30. Shi, W., Ling, D., Zhang, F., Fu, X., Lai, D., & Zhang, Y. (2021). Curcumin promotes osteogenic differentiation of human periodontal ligament stem cells by inducting EGR1 expression. Archives of oral biology, 121, 104958. https://doi.org/10.1016/j.archoralbio.2020.104958
31. Kameri, A., Dragidella, A., Haziri, A., Hashani, Z., Kurteshi, K., & Kurti, A. (2024). Antifungal and genotoxic effects of Thymus serpyllum as a root canal irrigant. Clinical and experimental dental research, 10(1), e837. https://doi.org/10.1002/cre2.837
32. Abdallah Khalil, A., & Alaaeldin, E. (2024). Sustained Release of Liposomal Curcumin: Enhanced Periodontal Outcomes in Diabetic Patients. The Chinese journal of dental research, 27(2), 169–174. https://doi.org/10.3290/j.cjdr.b5459607
33. Duane, B., Yap, T., Neelakantan, P., Anthonappa, R., Bescos, R., McGrath, C., McCullough, M., & Brookes, Z. (2023). Mouthwashes: Alternatives and Future Directions. International dental journal, 73 Suppl 2(Suppl 2), S89–S97. https://doi.org/10.1016/j.identj.2023.08.011
34. Takada, K., Nakano, S., Nishio, R., Muku, D., Mochizuki, S., Inui, I., Okita, K., Koga, A., Watanabe, K., Yoshioka, Y., Ariyoshi, W., & Yamasaki, R. (2024). Medicinal herbs, especially Hibiscus sabdariffa, inhibit oral pathogenic bacteria. Journal of oral biosciences, 66(1), 179–187. https://doi.org/10.1016/j.job.2024.01.006
35. Qamar, Z., Almohana, S. A., Khalid Alanazi, A., Khalid Alanazi, A., Almohana, A. A., & Zeeshan, T. (2021). Clinical Evaluation of the Effects of Topical Indocyanine-green Mediated Photosensitiser vs Aloe Vera Gel as Adjunct Therapy to Scaling and Root Planing in Chronic Periodontitis Patients. Oral health & preventive dentistry, 19, 489–494. https://doi.org/10.3290/j.ohpd.b2082037
36. Rutchanoo Chansamart, Polkit Sangvanich, Pasutha Thunyakitpisal,Clinical and Radiographic Evaluation of Combined Acemannan and Periodontal Surgery Induced-Periodontal Regeneration: 5-Year Follow-up Case Report,The Open Dentistry Journal,Volume 17,2023,ISSN 1874-2106,https://doi.org/10.2174/18742106-v17-e230124-2022-75.
37. Máximo, P. M., Cortelli, S. C., Aquino, D. R., de Miranda, T. B., Costa, F. O., & Cortelli, J. R. (2020). Preoperative Mouthwash in Subjects with Different Periodontal Status: A Randomised Controlled Clinical Trial. Oral health & preventive dentistry, 18(3), 433–440. https://doi.org/10.3290/j.ohpd.a44308
38. Wilder, R., Levine, W., & Paquette, D. W. (2022). Randomized Clinical Trial of a Topical Botanical Patch for the Adjunctive Management of Periodontitis. Oral health & preventive dentistry, 20, 253–262. https://doi.org/10.3290/j.ohpd.b3147141
39. Guzmán-Flores, J. M., Arevalo-Caro, C. M., Martínez-Esquivias, F., Isiordia-Espinoza, M. A., & Franco-de la Torre, L. (2023). Molecular mechanism of curcumin on periodontitis: A pharmacological network study. Journal of oral biosciences, 65(4), 379–385. https://doi.org/10.1016/j.job.2023.08.004
40. Pérez-Pacheco, C. G., Fernandes, N. A. R., Primo, F. L., Tedesco, A. C., Bellile, E., Retamal-Valdes, B., Feres, M., Guimarães-Stabili, M. R., & Rossa, C., Jr (2021). Local application of curcumin-loaded nanoparticles as an adjunct to scaling and root planing in periodontitis: Randomized, placebo-controlled, double-blind split-mouth clinical trial. Clinical oral investigations, 25(5), 3217–3227. https://doi.org/10.1007/s00784-020-03652-3

41. Rath, A., Wong, M., Li, K., Wong, A., Tan, L., Tan, K., & Pannuti, C. M. (2024). Efficacy of adjunctive octenidine hydrochloride as compared to chlorhexidine and placebo as adjuncts to instrumentation in stage I-II periodontitis: A double-blinded randomized controlled trial. International journal of dental hygiene, 22(4), 802–813. https://doi.org/10.1111/idh.12795
42. Maybodi, F. R., Herandi, V., & Vaezpour, M. S. (2025). Effect of aromatherapy with lemongrass (Cymbopogon citratus) on the anxiety of patients undergoing scaling and root planning: a randomized clinical trial. BMC complementary medicine and therapies, 25(1), 100. https://doi.org/10.1186/s12906-025-04834-w
43. Abullais, S. S., Patel, S. I., Asiri, E. A., Jathmi, A. A. A., Alkhayri, A. H., Mousa, Y. M., Ganem, A. A., & Mattoo, K. A. (2022). Comparative Evaluation of 3 Commercial Mouthwash Formulations on Clinical Parameters of Chronic Gingivitis. Medical science monitor : international medical journal of experimental and clinical research, 28, e937111. https://doi.org/10.12659/MSM.937111
44. Stańdo-Retecka, M., Piatek, P., Namiecinska, M., Bonikowski, R., Lewkowicz, P., & Lewkowicz, N. (2023). Clinical and microbiological outcomes of subgingival instrumentation supplemented with high-dose omega-3 polyunsaturated fatty acids in periodontal treatment - a randomized clinical trial. BMC oral health, 23(1), 290. https://doi.org/10.1186/s12903-023-03018-7
45. Yao, Y., Song, K., Chen, H., Ding, X., Shi, Q., Lu, X., & Cao, Y. (2021). In vitro and in vivo research of atmosphere pressure nonequilibrium plasmas on root canal disinfection: implication for alternative strategy for irrigation. Clinical oral investigations, 25(10), 5833–5842. https://doi.org/10.1007/s00784-021-03888-7
46. Çankaya, G., Ocak, M. S., Dündar, S., & Özercan, İ. H. (2025). Investigation of the relationship between apical periodontitis and host modulation provided by nutritional supplement: a study in rats. BMC oral health, 25(1), 336. https://doi.org/10.1186/s12903-025-05705-z
47. Manjunatha, V. A., Vemanaradhya, G. G., & Gowda, T. M. (2022). Clinical and antioxidant efficacy of 4% mangosteen gel as a local drug delivery in the treatment of chronic periodontitis: A placebo-controlled, split-mouth trial. Dental and medical problems, 59(1), 111–119. https://doi.org/10.17219/dmp/139198
48. Assiry, A. A., Karobari, M. I., Bhavikatti, S. K., & Marya, A. (2021). Crossover Analysis of the Astringent, Antimicrobial, and Anti-inflammatory Effects of Illicium verum/Star Anise in the Oral Cavity. BioMed research international, 2021, 5510174. https://doi.org/10.1155/2021/5510174
49. Kaplan, V., Hasanoglu Erbasar, G. N., Cigerim, L., Altay Turgut, H., & Cerit, A. (2021). Effect of St. John's wort oil and olive oil on the postoperative complications after third molar surgery: randomized, double-blind clinical trial. Clinical oral investigations, 25(4), 2429–2438. https://doi.org/10.1007/s00784-020-03639-0
50. Kim, Y. R., & Nam, S. H. (2022). A randomized, placebo-controlled clinical trial evaluating of a mouthwash containing Sambucus williamsii var. coreana extract for prevention of gingivitits. Scientific reports, 12(1), 11250. https://doi.org/10.1038/s41598-022-15445-7
51. De Rossi, A., Araújo Ferreira, D. C., Liévana, F. S., Vilela, M. M., Nelson-Filho, P., da Silva, R. S., Moraes, J. C. B., & da Silva, L. A. B. (2021). An Epigallocatechin-3-gallate Formulation Developed for Endodontic Use: A Physicochemical and Biological Evaluation. Journal of endodontics, 47(10), 1640–1650. https://doi.org/10.1016/j.joen.2021.06.010
52. Boyapati, R., Peeta, J., Dhulipalla, R., Kolaparthy, L., Adurty, C., & Cheruvu, R. N. S. (2024). Comparative evaluation of the efficacy of probiotic, Aloe vera, povidine-iodine, and chlorhexidine mouthwashes in the treatment of gingival inflammation: A randomized controlled trial. Dental and medical problems, 61(2), 181–190. https://doi.org/10.17219/dmp/156425

53. Pardiñas López, S., García-Caro, M. E., Vallejo, J. A., Aja-Macaya, P., Conde-Pérez, K., Nión-Cabeza, P., Khouly, I., Bou, G., Cendal, A. I. R., Díaz-Prado, S., & Poza, M. (2025). Anti-inflammatory and antimicrobial efficacy of coconut oil for periodontal pathogens: a triple-blind randomized clinical trial. Clinical oral investigations, 29(4), 182. https://doi.org/10.1007/s00784-025-06267-8
54. Al-Askar, M., AlMubarak, A. M., Alqutub, M. N., Mokeem, S., Javed, F., Vohra, F., & Abduljabbar, T. (2022). Analgesic Efficacy of Curcuma longa (Curcumin) after Surgical Periodontal Therapy. Oral health & preventive dentistry, 20, 19–26. https://doi.org/10.3290/j.ohpd.b2572979
55. Park, J. Y., Ko, K. A., Lee, J. Y., Oh, J. W., Lim, H. C., Lee, D. W., Choi, S. H., & Cha, J. K. (2021). Clinical and Immunological Efficacy of Mangosteen and Propolis Extracted Complex in Patients with Gingivitis: A Multi-Centered Randomized Controlled Clinical Trial. Nutrients, 13(8), 2604. https://doi.org/10.3390/nu13082604
56. Andhare, M. G., Shetty, S., Vivekanandan, G., Shetty, R. M., Rahman, B., Shetty, S. R., Siddeshappa, S. T., & Desai, V. (2024). Clinical efficacy of green tea, aloe vera and chlorhexidine mouthwashes in the treatment of dental biofilm induced gingivitis: A multi-arm, double-blinded, randomized controlled clinical trial. International journal of dental hygiene, 22(3), 504–513. https://doi.org/10.1111/idh.12664
57. Zuttion, G. S., Juárez, H. A. B., Lima, B. D., Assumpção, D. P., Daneris, Â. P., Tuchtenhagen, I. H., Casarin, M., & Muniz, F. W. M. G. (2024). Comparison of the anti-plaque and anti-gingivitis efficacy of Chlorhexidine and Malva mouthwashes: Randomized crossover clinical trial. Journal of dentistry, 150, 105313. https://doi.org/10.1016/j.jdent.2024.105313
58. Wasti, J., Wasti, A., & Singh, R. (2021). Efficacy of antioxidants therapy on progression of periodontal disease - A randomized control trial. Indian journal of dental research : official publication of Indian Society for Dental Research, 32(2), 187–191. https://doi.org/10.4103/ijdr.IJDR_227_20
59. Jung, J. S., Choi, G. H., Lee, H., Ko, Y., & Ji, S. (2024). The Clinical Effect of a Propolis and Mangosteen Extract Complex in Subjects with Gingivitis: A Randomized, Double-Blind, and Placebo-Controlled Clinical Trial. Nutrients, 16(17), 3000. https://doi.org/10.3390/nu16173000
60. Bayer, J., Petersen, N. K., Hess, J. V., Jockel-Schneider, Y., & Högger, P. (2025). Impact of a Dietary Supplementation with French Maritime Pine Bark Extract Pycnogenol® on Salivary and Serum Inflammatory Biomarkers During Non-Surgical Periodontal Therapy-A Randomized Placebo-Controlled Double-Blind Trial. Nutrients, 17(9), 1546. https://doi.org/10.3390/nu17091546
61. Kim, Y. R., & Nam, S. H. (2025). Effectiveness of Glycyrrhiza uralensis extract on periodontal pathogens: a randomized controlled clinical trial. BMC oral health, 25(1), 783. https://doi.org/10.1186/s12903-025-06172-2
62. Corbella, S., Radaelli, K., Alberti, A., Francetti, L., & Taschieri, S. (2024). Erythritol powder airflow for the treatment of peri-implant mucositis: A randomized controlled clinical trial. International journal of dental hygiene, 22(4), 982–990. https://doi.org/10.1111/idh.12814
63. Pérez-Pacheco, C. G., Fernandes, N. A. R., Primo, F. L., Tedesco, A. C., Bellile, E., Retamal-Valdes, B., Feres, M., Guimarães-Stabili, M. R., & Rossa, C., Jr (2021). Local application of curcumin-loaded nanoparticles as an adjunct to scaling and root planing in periodontitis: Randomized, placebo-controlled, double-blind split-mouth clinical trial. Clinical oral investigations, 25(5), 3217–3227. https://doi.org/10.1007/s00784-020-03652-3
64. Bakhshi, M., Mahboubi, A., Jaafari, M. R., Ebrahimi, F., Tofangchiha, M., & Alizadeh, A. (2022). COMPARATIVE EFFICACY OF 1% CURCUMIN NANOMICELLE GEL AND 2% CURCUMIN GEL FOR TREATMENT OF RECURRENT APHTHOUS STOMATITIS: A DOUBLE-BLIND RANDOMIZED CLINICAL TRIAL. The journal of evidence-based dental practice, 22(2), 101708. https://doi.org/10.1016/j.jebdp.2022.101708
65. Al-Mahmood, S., & Sabea, D. W. (2021). Comparative Evaluation of the Effectiveness of 40% Miswak Mouthwash and 0.12% Chlorhexidine Mouthwash in Treating Gingivitis: A Blinded, Randomised Clinical Trial. Oral health & preventive dentistry, 19, 229–233. https://doi.org/10.3290/j.ohpd.b1179501

66. Senkalvarayan, V., Kesavan, P., Dorairaj, J., Madhumala, R., Ravi, S., & Tomy, A. T. (2023). Comparative Evaluation of Efficacy of Herbal and Chlorhexidine Mouthwash on Gingival Health. Indian journal of dental research : official publication of Indian Society for Dental Research, 34(4), 401–404. https://doi.org/10.4103/ijdr.ijdr_293_22
67. Milleman, J., Bosma, M. L., McGuire, J. A., Sunkara, A., McAdoo, K., DelSasso, A., Wills, K., & Milleman, K. (2022). Comparative Effectiveness of Toothbrushing, Flossing and Mouthrinse Regimens on Plaque and Gingivitis: A 12-week virtually supervised clinical trial. Journal of dental hygiene : JDH, 96(3), 21–34.
68. Srikumar, K. P., Bhagyashree, B. N., Srirangarajan, S., Ravi, R. J., & Vinaya, R. (2022). Efficacy of Melaleuca alternifolia and chlorhexidine mouth rinses in reducing oral malodor and Solobacterium moorei levels. A 1 week, randomized, double-blind, parallel study. Indian journal of pharmacology, 54(2), 77–83. https://doi.org/10.4103/ijp.ijp_772_20
69. Erbasar, G. N. H., Kaplan, V., Cigerim, L., Konarili, F. N., & Sahin, M. (2023). Effect of combined boric acid and chlorhexidine mouthwashes on postoperative complications and periodontal healing after impacted third molar surgery: a-double blind randomized study. Clinical oral investigations, 27(7), 3817–3826. https://doi.org/10.1007/s00784-023-04999-z
70. Al-Zawawi, A. S., Shaheen, M. Y., Divakar, D. D., Aldulaijan, H. A., & Basudan, A. M. (2022). Postoperative anti-inflammatory efficacy of 2% saline rinses and a herbal- mouthwash after non-surgical periodontal therapy for the management of periodontal inflammation in young adults with chlorhexidine allergy: A randomized controlled trial. International journal of dental hygiene, 20(2), 408–414. https://doi.org/10.1111/idh.12583
71. Kamath, D. G., Nadimpalli, H., Nayak, S. U., Rajendran, V., & Natarajan, S. (2023). Comparison of antiplaque and anti-gingivitis effects of aloe vera mouthwash with chlorhexidine in fixed orthodontic patients-A randomized controlled trial. International journal of dental hygiene, 21(1), 211–218. https://doi.org/10.1111/idh.12615
72. Alqutub, M. N., Alhumaidan, A. A., Alali, Y., Al-Aali, K. A., Javed, F., Vohra, F., & Abduljabbar, T. (2023). Comparison of the postoperative anti-inflammatory efficacy of chlorhexidine, saline rinses and herbal mouthwashes after mechanical debridement in patients with peri-implant mucositis: A randomized controlled trial. International journal of dental hygiene, 21(1), 203–210. https://doi.org/10.1111/idh.12582
73. Sahu, S. A., Panda, S., Das, A. C., Mishra, L., Rath, S., Sokolowski, K., Kumar, M., Mohanty, R., Nayak, R., Satpathy, A., & Lapinska, B. (2023). Efficacy of Sub-Gingivally Delivered Propolis Nanoparticle in Non-Surgical Management of Periodontal Pocket: A Randomized Clinical Trial. Biomolecules, 13(11), 1576. https://doi.org/10.3390/biom13111576

74. Amano, S., Matsumoto, M., Morimoto, M., Kawamoto, H., Takeshita, F., Yasui, T., & Sakagami, H. (2025). Efficacy of toothpaste containing Brazilian green propolis extracts with an optimal kaempferide/betuletol ratio for improving oral microbiota: A randomized, controlled, paired crossover study. Journal of ethnopharmacology, 337(Pt 1), 118762. https://doi.org/10.1016/j.jep.2024.118762
75. Abdel-Fatah, R., Mowafey, B., Baiomy, A., & Elmeadawy, S. (2023). Efficacy of curcumin gel as an adjunct to scaling and root planing on salivary procalcitonin level in the treatment of patients with chronic periodontitis: a randomized controlled clinical trial. BMC oral health, 23(1), 883. https://doi.org/10.1186/s12903-023-03512-y
76. Sutthiboonyapan, P., Sriratanasak, N., Innets, B., Angkanaporn, N., Suntornchot, P., Panyain, W., Porntaveetus, T., Wiriyakijja, P., & Chanvorachote, P. (2025). A Randomized Double-Blind Controlled Evaluation of the Therapeutic Benefits of an Herbal Lip Hydrant. Journal of cosmetic dermatology, 24(3), e70041. https://doi.org/10.1111/jocd.70041

77. Eltantawi, AR, Abdel-Razik, GM, Elhawary, YM e Badr, AE (2024). Efficacia della glicirrizina come farmaco intracanalare sulla riduzione della carica batterica nei canali radicolari infetti primari: uno studio clinico randomizzato. The Journal of Contemporary Dental Practice , 25 (6), 540-546.
78. Kiani, S., Birang, R., & Jamshidian, N. (2022). Effect of Propolis mouthwash on clinical periodontal parameters in patients with gingivitis: A double-blinded randomized clinical trial. International journal of dental hygiene, 20(2), 434–440. https://doi.org/10.1111/idh.12550
79. Valkenburg, C., Rosema, N. A. M., Hennequin-Hoenderdos, N. L., Versteeg, P. A., Slot, D. E., & Van der Weijden, G. A. F. (2021). Do natural ingredients in a dentifrice contribute to prevention of plaque and gingivitis?. International journal of dental hygiene, 19(4), 429–439. https://doi.org/10.1111/idh.12517
80. Qamar, Z., Almohana, S. A., Khalid Alanazi, A., Khalid Alanazi, A., Almohana, A. A., & Zeeshan, T. (2021). Clinical Evaluation of the Effects of Topical Indocyanine-green Mediated Photosensitiser vs Aloe Vera Gel as Adjunct Therapy to Scaling and Root Planing in Chronic Periodontitis Patients. Oral health & preventive dentistry, 19, 489–494. https://doi.org/10.3290/j.ohpd.b2082037
81. Katanasaka, Y., Yoshida, N., Naitou, H., Naruta, R., Miyazaki, Y., Sunagawa, Y., Funamoto, M., Shimizu, K., Shimizu, S., Sari, N., Yamakage, H., Satoh-Asahara, N., Hasegawa, K., & Morimoto, T. (2021). Effect of Theaflavin on Oral Bacteria in Japanese Subjects: A Randomized, Placebo-Controlled, Double-Blind Study. Journal of medicinal food, 24(11), 1186–1190. https://doi.org/10.1089/jmf.2021.K.0050
82. Khabazian, A., Mirhashemi, F. S., & Sadeghi, F. (2025). Investigating the effect of propolis-containing chewing gum in comparison with propolis-containing mouthwash on reducing gingival inflammation in patients with gingivitis. BMC oral health, 25(1), 231. https://doi.org/10.1186/s12903-025-05564-8
83. Bapat, R. A., Bedia, S. V., Bedia, A. S., Yang, H. J., Dharmadhikari, S., Abdulla, A. M., Chaubal, T. V., Bapat, P. R., Abullais, S. S., Wahab, S., & Kesharwani, P. (2023). Current appraises of therapeutic applications of nanocurcumin: A novel drug delivery approach for biomaterials in dentistry. Environmental research, 238(Pt 1), 116971. https://doi.org/10.1016/j.envres.2023.116971
84. Abdul-Wahab, H. Y., Salah, R., & Abdulbaqi, H. R. (2025). Salivary levels of catalase, total antioxidant capacity and interleukin-1β and oral health-related quality of life after matcha and green tea consumption for patients with gingivitis: A randomized clinical trial. International journal of dental hygiene, 23(1), 114–123. https://doi.org/10.1111/idh.12820
85. González-Serrano, J., López-Pintor, R. M., Serrano, J., Torres, J., Hernández, G., & Sanz, M. (2021). Short-term efficacy of a gel containing propolis extract, nanovitamin C and nanovitamin E on peri-implant mucositis: A double-blind, randomized, clinical trial. Journal of periodontal research, 56(5), 897–906. https://doi.org/10.1111/jre.12886
86. Cosola, S., Oldoini, G., Giammarinaro, E., Covani, U., Genovesi, A., & Marconcini, S. (2022). The effectiveness of the information-motivation model and domestic brushing with a hypochlorite-based formula on peri-implant mucositis: A randomized clinical study. Clinical and experimental dental research, 8(1), 350–358. https://doi.org/10.1002/cre2.487
87. Kim, Y. R., & Nam, S. H. (2025). Effects of oral hygiene management containing Cibotium Barometz J. Smith extract on peri-implant mucositis: a randomized clinical trial. BMC complementary medicine and therapies, 25(1), 164. https://doi.org/10.1186/s12906-025-04900-3
88. Taalab, M. R., Mahmoud, S. A., Moslemany, R. M. E., & Abdelaziz, D. M. (2021). Intrapocket application of tea tree oil gel in the treatment of stage 2 periodontitis. BMC oral health, 21(1), 239. https://doi.org/10.1186/s12903-021-01588-y
89. Azizan, N. F., Mohd, N., Nik Azis, N. M., & Baharin, B. (2023). Effectiveness of Salvadora persica toothbrush and Salvadora persica chewing stick in plaque and gingivitis control: a randomized control trial. BMC complementary medicine and therapies, 23(1), 456. https://doi.org/10.1186/s12906-023-04295-z

90. Chaganti, S., Kunthsam, V., Velangini, S. Y., Alzahrani, K. J., Alzahrani, F. M., Halawani, I. F., Alshahrani, M., Ashi, H., Baeshen, H. A., & Patil, S. (2023). Comparison of bacterial colonization on absorbable non-coated suture with Triclosan- or Chlorhexidine-coated sutures: a randomized controlled study. European review for medical and pharmacological sciences, 27(18), 8371–8383. https://doi.org/10.26355/eurrev_202309_33760
91. Bolla, V. L., Jyothi, M., Mettu, S. R., Manoj Kumar, M. G., Rao, K. N., Reddy, M. S., & Koppolu, P. (2023). Effectiveness of three mouth rinsing agents against mutans Streptococcus and Lactobacillus species - A comparative study. Annals of African medicine, 22(3), 365–372. https://doi.org/10.4103/aam.aam_95_22
92. Loaiza Oliva, M., Morales Uchima, S. M., Puerta Suárez, J., Mesa Arango, A. C., & Martínez Pabón, M. C. (2023). Lippia origanoides derivatives in vitro evaluation on polymicrobial biofilms: Streptococcus mutans, Lactobacillus rhamnosus and Candida albicans. Archives of oral biology, 148, 105656. https://doi.org/10.1016/j.archoralbio.2023.105656
93. Hussein, H., & Kishen, A. (2022). Local Immunomodulatory Effects of Intracanal Medications in Apical Periodontitis. Journal of endodontics, 48(4), 430–456. https://doi.org/10.1016/j.joen.2022.01.003
94. Derman, S. H. M., Lantwin, E. M., Barbe, A. G., & Noack, M. J. (2021). Does a pretreatment with a dentine hypersensitivity mouth-rinse compensate the pain caused by professional mechanical plaque removal? A single-blind randomized controlled clinical trial. Clinical oral investigations, 25(5), 3151–3160. https://doi.org/10.1007/s00784-020-03643-4
95. Sayar, F., Farahmand, A. H., & Rezazadeh, M. (2021). Clinical Efficacy of Aloe Vera Toothpaste on Periodontal Parameters of Patients with Gingivitis-A Randomized, Controlled, Single-masked Clinical Trial. The journal of contemporary dental practice, 22(3), 242–247.
96. Eltay, E. G., Gismalla, B. G., Mukhtar, M. M., & Awadelkarim, M. O. A. (2021). Punica granatum peel extract as adjunct irrigation to nonsurgical treatment of chronic gingivitis. Complementary therapies in clinical practice, 43, 101383. https://doi.org/10.1016/j.ctcp.2021.101383
97. Vitiello, F., Monterubbianesi, R., Sparabombe, S., Bourgeois, D., Tosco, V., Alshehri, F. A., Carrouel, F., Putignano, A., & Orsini, G. (2024). Use of over-the-counter mouthwashes as an additional measure in individual oral prophylaxis on adults with plaque-induced gingivitis: a double-blind, parallel, randomized controlled trial. BMC oral health, 24(1), 83. https://doi.org/10.1186/s12903-023-03779-1
98. Potewiratnanond, P., Surarit, R., Tantisira, M. H., Samaranayake, L., Rotpenpian, N., & Wanasuntronwong, A. (2025). Efficacy of Centella asiatica on mitigating temporomandibular pain and improving functionality: a randomized, double blind, pilot clinical trial. Head & face medicine, 21(1), 28. https://doi.org/10.1186/s13005-025-00503-y
99. Laky, B., Bruckmann, C., Blumenschein, J., Durstberger, G., & Haririan, H. (2024). Effect of a multinutrient supplement as an adjunct to nonsurgical treatment of periodontitis: A randomized placebo-controlled clinical trial. Journal of periodontology, 95(2), 101–113. https://doi.org/10.1002/JPER.23-0115
100. Stańdo-Retecka, M., Piatek, P., Namiecinska, M., Bonikowski, R., Lewkowicz, P. e Lewkowicz, N. (2023). Risultati clinici e microbiologici della strumentazione sottogengivale integrata con acidi grassi polinsaturi omega-3 ad alto dosaggio nel trattamento parodontale: uno studio clinico randomizzato. BMC Oral Health , 23 (1), 290.
101. Matos, Y. R., de Carvalho Leal, I., da Silva, A. B. A., de Oliveira, N. F., Pinheiro, I. R., Veras, P. M. O., de Barros Silva, P. G., Sampaio, E. F., & Cetira Filho, E. L. (2024). Effect of chloramine T and chlorhexidine in reducing gingivitis in patients admitted to a reference hospital for cardiopulmonary care: a randomized, controlled clinical trial. Clinical oral investigations, 28(11), 619. https://doi.org/10.1007/s00784-024-06012-7
102. Fan, Q., Zhou, X. H., Wang, T. F., Zeng, F. J., Liu, X., Gu, Y., Chen, B., Yang, J., Pang, Z. Y., Liu, J. G., & Bai, G. H. (2023). Effects of epigallocatechin-3-gallate on oxidative stress, inflammation, and bone loss in a rat periodontitis model. Journal of dental sciences, 18(4), 1567–1575. https://doi.org/10.1016/j.jds.2023.02.019

103. Yaghmoor, W., Ruiz-Torruella, M., Ogata, Y., Natto, Z. S., Finkelman, M., Kawai, T., & Hur, Y. (2024). Effect of preoperative chlorhexidine, essential oil, and cetylpyridinium chloride mouthwashes on bacterial contamination during dental implant surgery: A randomized controlled clinical trial. The Saudi dental journal, 36(3), 492–497. https://doi.org/10.1016/j.sdentj.2023.12.011
104. Fahim, M. F. M., & Zarnigar, P. (2024). Effect of prepared herbal mouthwash in maintaining the oral health of school children: A single-blind randomised control trial. Explore (New York, N.Y.), 20(4), 535–543. https://doi.org/10.1016/j.explore.2023.12.003
105. Weber, J., Scholz, K. J., Schenke, I. M., Pfab, F., Cieplik, F., Hiller, K. A., Buchalla, W., Sahm, C., Kirschneck, C., & Paddenberg-Schubert, E. (2024). Randomized controlled clinical trial on the efficacy of a novel antimicrobial chewing gum in reducing plaque and gingivitis in adolescent orthodontic patients. Clinical oral investigations, 28(5), 272. https://doi.org/10.1007/s00784-024-05669-4
106. Mathew, C. A., Veena, H. R., Shubha, P., & Daniel, R. A. (2023). Antimicrobial photocatalysis using bio-hydrothermally synthesized Zinc oxide nanoparticles in the management of periodontitis: a prospective split-mouth, double-blind, randomized, controlled clinical trial. Journal of applied oral science : revista FOB, 31, e20230271. https://doi.org/10.1590/1678-7757-2023-0271
107. Nandlal, B., Sreenivasan, P. K., Shashikumar, P., Devishree, G., & Bettahalli Shivamallu, A. (2021). A randomized clinical study to examine the oral hygiene efficacy of a novel herbal toothpaste with zinc over a 6-month period. International journal of dental hygiene, 19(4), 440–449. https://doi.org/10.1111/idh.12505
108. Jockel-Schneider, Y., Stölzel, P., Endres, J., Petersen, N., Haubitz, I., Heß, J., & Schlagenhauf, U. (2024). Impact of the uninstructed use of a herbal, ayurvedic toothpaste on parameters of gingival health in periodontal aftercare patients: A randomized, double-blinded, two-arm parallel-group study. International journal of dental hygiene, 22(3), 647–654. https://doi.org/10.1111/idh.12743
109. Birjandi, A. A., & Sharpe, P. (2025). Therapeutic potential of curcumin in regenerative dentistry. Frontiers in dental medicine, 6, 1537478. https://doi.org/10.3389/fdmed.2025.1537478
110. Deesricharoenkiat, N., Jansisyanont, P., Chuenchompoonut, V., Mattheos, N., & Thunyakitpisal, P. (2022). The effect of acemannan in implant placement with simultaneous guided bone regeneration in the aesthetic zone: a randomized controlled trial. International journal of oral and maxillofacial surgery, 51(4), 535–544. https://doi.org/10.1016/j.ijom.2021.07.017
111. Zhang, P., Chen, S. G., Wang, J. T., Wang, J. D., Chen, Z. H., & Lin, H. S. (2024). A study on the impact of gargling with compound Scutellaria baicalensis Georgi on oral health and microflora changes in fixed orthodontic patients: An experimental study. Medicine, 103(34), e39397. https://doi.org/10.1097/MD.0000000000039397
112. Chaubal, T. V., Ywen, B. S., Ying Ying, T., & Bapat, R. (2024). Clinical and microbiologic effect of local application of curcumin as an adjunct to scaling and root planing in periodontitis: Systematic review. Irish journal of medical science, 193(4), 1985–1994. https://doi.org/10.1007/s11845-024-03635-3
113. Abdallah Khalil, A., & Alaaeldin, E. (2024). Sustained Release of Liposomal Curcumin: Enhanced Periodontal Outcomes in Diabetic Patients. The Chinese journal of dental research, 27(2), 169–174. https://doi.org/10.3290/j.cjdr.b5459607
114. Zandesh, A., Mehrpouya, M., Panahi, P., Motallebi, N., Sharifi, S., Dizaj, S. M., & Ghavimi, M. A. (2025). A Split-Mouth, Randomized, Double-Blind Clinical Trial of a Gelatin Hemostatic Dental Sponge Containing Aloe Vera Nanoparticles for Controlling Bleeding After Mandibular Posterior Teeth Extraction. Current pharmaceutical design, 31(41), 3312–3318. https://doi.org/10.2174/0113816128363906250407111215

115. Baltieri, P. W. Q., de Araújo, L. P., Gomes, B. P. F. A., de Almeida, J. F. A., Ferraz, C. C. R., & de-Jesus-Soares, A. (2024). Outcome of Nonsurgical Root Canal Retreatment of Teeth with Persistent Apical Periodontitis Treated with Foraminal Enlargement and 2% Chlorhexidine Gel: A Retrospective Cohort Study. Journal of endodontics, 50(11), 1551–1559. https://doi.org/10.1016/j.joen.2024.09.006
116. Talasani, R. R., Potharaju, S. P., Vijaya Lakshmi, B., Durga Bai, Y., Chintala, R. K., Mahankali, V., Koppolu, P., & AlGhamdi, A. R. S. (2022). Efficacy of ozonated water over chlorhexidine mouth rinse in chronic gingivitis patients - A comparative clinical study. The Saudi dental journal, 34(8), 738–743. https://doi.org/10.1016/j.sdentj.2022.09.004
117. Kose, O., Sarac Gul, Y., Altin, A., Bostan, S. A., Faiz, O., Akyildiz, K., & Yilmaz, A. (2025). Clinical and Biochemical Efficacies of Green and White Tea Extract Mouthwashes in the Management of Plaque-Induced Gingivitis. International journal of dental hygiene, 23(3), 482–490. https://doi.org/10.1111/idh.12890
118. Kamath, D. G., Nadimpalli, H., Nayak, S. U., Rajendran, V., & Natarajan, S. (2023). Comparison of antiplaque and anti-gingivitis effects of aloe vera mouthwash with chlorhexidine in fixed orthodontic patients-A randomized controlled trial. International journal of dental hygiene, 21(1), 211–218. https://doi.org/10.1111/idh.12615
119. Huang, Y. F., Yang, H. W., & Lin-Shiau, S. Y. (2022). Novel regimens of phytopolyphenols with cisplatin or memantine and $ZnSO_4$ for synergistic inhibition of growth and gingipains of the cultured Porphyromonas gingivalis. Journal of dental sciences, 17(4), 1796–1801. https://doi.org/10.1016/j.jds.2022.06.015
120. ALMUTAIRI, A. S. (2022). GENERAL DENTISTS'AWARENESS ON CHLORHEXIDINE HYPERSENSITIVITY IN SAUDI ARABIA. A CROSS-SECTIONAL STUDY. International Journal of Medical Dentistry, 26(2).
121. Langa, G. P. J., Cavagni, J., Muniz, F. W. M. G., Oballe, H. J. R., Friedrich, S. A., Nicolini, A. C., Thomé, D. P., Sossai, L. L., Rup, A. G., Malheiros, Z., Stewart, B., Kilpatrick, L., Ryan, M., & Rösing, C. K. (2021). Antiplaque and antigingivitis efficacy of cetylpyridinium chloride with zinc lactate compared with essential oil mouthrinses: Randomized clinical trial. Journal of the American Dental Association (1939), 152(2), 105–114. https://doi.org/10.1016/j.adaj.2020.09.021
122. Lorusso, F., Tartaglia, G., Inchingolo, F., & Scarano, A. (2022). Early Response and Clinical Efficacy of a Mouthwash Containing Chlorhexidine, Anti Discoloration System, Polyvinylpyrrolidone/Vinyl Acetate and Sodium DNA in Periodontitis Model: A Triple-Blind Randomized Controlled Clinical Trial. Dentistry journal, 10(6), 101. https://doi.org/10.3390/dj10060101
123. Hermes, S., Alatwan, S., Johansson, A. e Bogren, A. (2024). Efficacia degli agenti terapeutici naturali nelle strategie preventive contro il patogeno parodontale Aggregatibacter actinomycetemcomitans : uno studio in vitro. Orale , 4 (3), 405-416. https://doi.org/10.3390/oral4030033
124. Manohar Sharma, H.1 , ; Deepika, PC1 ; Venkatesh, deputato 1 ; Chandan, S.2 ; Shashikumar, Pratibha 1 .Efficacia della somministrazione locale di Psidium Guajava al 3% nel trattamento della parodontite cronica: Uno studio clinico randomizzato controllato. Journal of International Oral Health 13(1):p 17-23, gennaio-febbraio 2021. | DOI: 10.4103/jioh.jioh_249_20
125. Brookes, Z., McGrath, C., & McCullough, M. (2023). Antimicrobial Mouthwashes: An Overview of Mechanisms-What Do We Still Need to Know?. International dental journal, 73 Suppl 2(Suppl 2), S64–S68. https://doi.org/10.1016/j.identj.2023.08.009
126. Moradi, S., Moushekhian, S., Najafi, E., Sedigh, H. S., & Navabi, S. (2023). Efficacy of propolis and triple antibiotic paste as intra-canal medicaments for revascularisation of immature teeth in dogs: a comparative study. European archives of paediatric dentistry : official journal of the European Academy of Paediatric Dentistry, 24(3), 321–326. https://doi.org/10.1007/s40368-023-00806-1

127. Al-Abdullah, A., Edris, S., Abu Hasna, A., de Carvalho, L. S., & Al-Nahlawi, T. (2022). The Effect of Aloe vera and Chlorhexidine as Disinfectants on the Success of Selective Caries Removal Technique: A Randomized Controlled Trial. International journal of dentistry, 2022, 9474677. https://doi.org/10.1155/2022/9474677
128. Hameeda P, Katti S, Jammalamadugu R, Bhatt K, Peram MR, Kumbar V. Confronto degli effetti della curcumina e della nanocurcumina sulla sopravvivenza delle cellule staminali mesenchimali derivate dall'uomo: uno studio sperimentale. Journal of Advanced Oral Research . 2020;11(2):148-155. doi: 10.1177/2320206820949741
129. Souza, B. D. M., Garcia, L. F. R., Bortoluzzi, E. A., Felippe, W. T., & Felippe, M. C. S. (2020). Effects of several storage media on viability and proliferation capacity of periodontal ligament cells. European archives of paediatric dentistry : official journal of the European Academy of Paediatric Dentistry, 21(1), 53–59. https://doi.org/10.1007/s40368-019-00450-8
130. Lan, C., Qian, Y., Wang, Y., Chen, Y., Lin, C., Zhang, Y., & Huang, X. (2023). The protective role of curcumin in human dental pulp stem cells stimulated by lipopolysaccharide via inhibiting NF-κB p65 phosphorylation to suppress NLRP3 inflammasome activation. Clinical oral investigations, 27(6), 2875–2885. https://doi.org/10.1007/s00784-023-04885-8
131. Nam, O. H., Ro, S. T., Lee, H. W., Jeong, J., Chae, Y. K., Lee, K. E., Choi, S. C., & Kang, S. W. (2023). Evaluation of delphinidin as a storage medium for avulsed teeth. BMC oral health, 23(1), 21. https://doi.org/10.1186/s12903-023-02713-9
132. Marconcini, Simone 1 ; Cosola, Saverio 2 ; Giammarinaro, Enrica 2, ; Oldoini, Giacomo1 ; Genovesi, Annamaria 3 .Efficacia di un approccio incrementale alla terapia parodontale non chirurgica con l'uso di una formulazione aggiuntiva di ipoclorito allo 0,02% a rilascio lento somministrata localmente: Uno studio clinico randomizzato. Rivista di salute orale internazionale 15(4):p 350-356, luglio-agosto 2023. | DOI: 10.4103/jioh.jioh_269_22
133. Saini, R. S., Vaddamanu, S. K., Dermawan, D., Bavabeedu, S. S., Khudaverdyan, M., Mosaddad, S. A., & Heboyan, A. (2025). In Silico Docking of Medicinal Herbs Against P. gingivalis for Chronic Periodontitis Intervention. International dental journal, 75(2), 1113–1135. https://doi.org/10.1016/j.identj.2024.06.019
134. Stanton, K. A., & McCracken, B. A. (2021). An activated-zinc oral rinse reduces pro-inflammatory cytokine secretion and promotes proliferation in Porphyromonas gingivalis LPS-challenged gingival tissues - A pilot study. Clinical and experimental dental research, 7(6), 995–1001. https://doi.org/10.1002/cre2.437
135. Dai, Q., Lee, H. M., Giordano, A., Chiang, F. P., Walker, S. G., Delgado-Ruiz, R., Johnson, F., Golub, L. M., & Gu, Y. (2025). Effect of a chemically-modified-curcumin on dental resin biodegradation. Frontiers in oral health, 5, 1506616. https://doi.org/10.3389/froh.2024.1506616
136. Bello, L., Romano, F., Gaido, C., & Defabianis, P. (2020). The effect of an oral spray containing an aqueous extract of Triticum vulgare on dental plaque and gingival inflammation in schoolchildren: A randomized controlled trial. European journal of paediatric dentistry, 21(2), 110–114. https://doi.org/10.23804/ejpd.2020.21.02.04
137. Noushad, M. C., Ashraf, K., & Suneetha, M. P. (2020). Antibacterial Efficacy of Muringa Seed Extract and Potato Peel Extract Against Enterococcus faecalis. Contemporary clinical dentistry, 11(4), 327–331. https://doi.org/10.4103/ccd.ccd_223_19
138. Lisda Damayanti, Ida Ayu Evaangelina e Avi Laviana et al. Attività antibatterica di Buah Merah (Pandanus conoideus Lam.) contro il patogeno batterico orale di Streptococcus sanguinis ATCC10556, Streptococcus mutans ATCC 25175 e Enterococcus faecalis ATCC 29212: uno studio in vitro. Il giornale aperto di odontoiatria. 2020. vol. 14(1):113-119. DOI: 10.2174/18742106020140113

139. Ji, L. L., Song, G., Jiang, L. M., Liu, Y., Ding, Z. J., Zhuang, X. Y., & Chen, X. (2021). Evaluation of conditioned medium from placenta-derived mesenchymal stem cells as a storage medium for avulsed teeth: An in vitro study. Dental traumatology : official publication of International Association for Dental Traumatology, 37(1), 73–80. https://doi.org/10.1111/edt.12599
140. Aherne, O., Ortiz, R., Fazli, M. M., & Davies, J. R. (2022). Effects of stabilized hypochlorous acid on oral biofilm bacteria. BMC oral health, 22(1), 415. https://doi.org/10.1186/s12903-022-02453-2
141. Rawat, Aditi; Kaur, Navpreet; Priya, Ratna; Sharma, Vivek; Bhalla, Manish; Gupta, Roopali .Valutazione dell'efficacia antiplacca di un dentifricio a base di erbe e non a base di erbe: uno studio clinico randomizzato. Rivista dell'Associazione indiana di odontoiatria sanitaria pubblica 22(2):p 148-153, aprile-giugno 2024. | DOI: 10.4103/jiaphd.jiaphd_227_22
142. Rodrigues, G. W. L., Del Bianco Vargas Gouveia, S., Oliveira, L. C., de Freitas, R. N., Dourado, N. G., Sacoman, C. A., Ribeiro, A. P. F., Chaves-Neto, A. H., Sivieri-Araújo, G., de Toledo Leonardo, R., Cintra, L. T. A., & Jacinto, R. C. (2025). Comparative analysis of antimicrobial activity and oxidative damage induced by laser ablation with indocyanine green versus aPDT with methylene blue and curcumin on E. coli biofilm in root canals. Odontology, 10.1007/s10266-025-01103-7. Advance online publication. https://doi.org/10.1007/s10266-025-01103-7
143. Herdiyati, Y., Atmaja, H. E., Satari, M. H., & Kurnia, D. (2020). Potential Antibacterial Flavonoid from Buah Merah (Pandanus conodieus Lam.) Against Pathogenic Oral Bacteria of Enterococcus faecalis ATCC 29212. The Open Dentistry Journal, 14(1).
144. Bunwanna, A., Damrongrungruang, T., Puasiri, S., Kantrong, N., & Chailertvanitkul, P. (2021). Preservation of the viability and gene expression of human periodontal ligament cells by Thai propolis extract. Dental traumatology : official publication of International Association for Dental Traumatology, 37(1), 123–130. https://doi.org/10.1111/edt.12612
145. Kommuri, K., Michelogiannakis, D., Barmak, B. A., Rossouw, P. E., & Javed, F. (2022). Efficacy of herbal- versus chlorhexidine-based mouthwashes towards oral hygiene maintenance in patients undergoing fixed orthodontic therapy: A systematic review and meta-analysis. International journal of dental hygiene, 20(1), 100–111. https://doi.org/10.1111/idh.12567
146. Rodrigues, MTV, Guillen, GA, Macêdo, FGC, Goulart, DR e Nóia, CF (2023). Effetti comparativi di diversi materiali sulla conservazione alveolare. Giornale di chirurgia orale e maxillofacciale , 81 (2), 213-223.
147. Deesricharoenkiat, N., Jansisyanont, P., Chuenchompoonut, V., Mattheos, N. e Thunyakitpisal, P. (2022). L'effetto dell'acemannano nel posizionamento dell'impianto con rigenerazione ossea guidata simultanea nella zona estetica: uno studio randomizzato controllato. International Journal of Oral and Maxillofacial Surgery , 51 (4), 535-544

148. Cheng, C. Y., Chen, Y. H., Thuy Tien Vo, T., Chui Hong, Y., Wang, C. S., Canh Vo, Q., Chou, H. C., Huang, T. W., & Lee, I. T. (2022). CORM-2 prevents human gingival fibroblasts from lipoteichoic acid-induced VCAM-1 and ICAM-1 expression by inhibiting TLR2/MyD88/TRAF6/PI3K/Akt/ROS/NF-κB signaling pathway. Biochemical pharmacology, 201, 115099. https://doi.org/10.1016/j.bcp.2022.115099
149. Taniguchi, Y., Ouhara, K., Sato, Y., Shoji, M., Hou, Y., Zhai, R., Fujimori, R., Kuwahara, N., Tamura, T., Matsuda, S., & Mizuno, N. (2025). Suppressive Effects of Kouboku on Methyl Mercaptan Production and Biofilm Formation in Porphyromonas gingivalis. Molecular oral microbiology, 40(3), 128–136. https://doi.org/10.1111/omi.12493
150. Eltantawi, AR, Abdel-Razik, GM, Elhawary, YM e Badr, AE (2024). Efficacia della glicirrizina come farmaco intracanalare sulla riduzione della carica batterica nei canali radicolari infetti primari: uno studio clinico randomizzato. The Journal of Contemporary Dental Practice , 25 (6), 540-546.

151. Garcia, C. R., Ueda, T. Y., da Silva, R. A., Cano, I. P., Saldanha, L. L., Dokkedal, A. L., Porto, V. C., Urban, V. M., & Neppelenbroek, K. H. (2022). Effect of denture liners surface modification with Equisetum giganteum and Punica granatum on Candida albicans biofilm inhibition. Therapeutic delivery, 13(3), 157–166. https://doi.org/10.4155/tde-2021-0074
152. Ghavimi, M. A., Bani Shahabadi, A., Jarolmasjed, S., Memar, M. Y., Maleki Dizaj, S., & Sharifi, S. (2020). Nanofibrous asymmetric collagen/curcumin membrane containing aspirin-loaded PLGA nanoparticles for guided bone regeneration. Scientific reports, 10(1), 18200. https://doi.org/10.1038/s41598-020-75454-2
153. Zhang, C., Jahan, S. A., Zhang, J., Bianchi, M. B., Volpe-Zanutto, F., Baviskar, S. M., Rodriguez-Abetxuko, A., Mishra, D., Magee, E., Gilmore, B. F., Singh, T. R. R., Donnelly, R. F., Larrañeta, E., & Paredes, A. J. (2023). Curcumin nanocrystals-in-nanofibres as a promising platform for the management of periodontal disease. International journal of pharmaceutics, 648, 123585. https://doi.org/10.1016/j.ijpharm.2023.123585
154. Abullais Saquib, S., Abdullah AlQahtani, N., Ahmad, I., Arora, S., Mohammed Asif, S., Ahmed Javali, M., & Nisar, N. (2021). Synergistic antibacterial activity of herbal extracts with antibiotics on bacteria responsible for periodontitis. Journal of infection in developing countries, 15(11), 1685–1693. https://doi.org/10.3855/jidc.14904
155. Strappa, EM, Meme, L., Cerea, M., Roy, M., & Bambini, F. (2022). Impianto sottoperiosteo realizzato su misura con tecnica additiva. Minerva dental and oral science , 71 (6), 353-360.
156. Grant, M. M., Scott, A. E., Matthews, J. B., Griffiths, H. R., & Chapple, I. L. C. (2023). Pre-conditioning of gingival epithelial cells with sub-apoptotic concentrations of curcumin prevents pro-inflammatory cytokine release. Journal of periodontal research, 58(3), 634–645. https://doi.org/10.1111/jre.13114
157. Calabrese E. J. (2021). Human periodontal ligament stem cells and hormesis: Enhancing cell renewal and cell differentiation. Pharmacological research, 173, 105914. https://doi.org/10.1016/j.phrs.2021.105914
158. Scott, J., & Marshman, Z. (2022). Does the use of miswak reduce plaque and gingivitis among adults?. Evidence-based dentistry, 23(4), 152–153. https://doi.org/10.1038/s41432-022-0833-y
159. Zuttion, G. S., Juárez, H. A. B., Lima, B. D., Assumpção, D. P., Daneris, Â. P., Tuchtenhagen, I. H., Casarin, M., & Muniz, F. W. M. G. (2024). Comparison of the anti-plaque and anti-gingivitis efficacy of Chlorhexidine and Malva mouthwashes: Randomized crossover clinical trial. Journal of dentistry, 150, 105313. https://doi.org/10.1016/j.jdent.2024.105313
160. Vo, T. T. T., Lee, C. W., Chiang, Y. C., Chen, Y. W., Yu, Y. H., Tuan, V. P., Wu, C. Z., & Lee, I. T. (2021). Protective mechanisms of Taiwanese green propolis toward high glucose-induced inflammation via NLRP3 inflammasome signaling pathway in human gingival fibroblasts. Journal of periodontal research, 56(4), 804–818. https://doi.org/10.1111/jre.12879
161. Yoshida, S., Inaba, H., Nomura, R., Nakano, K., & Matsumoto-Nakano, M. (2022). Green tea catechins inhibit Porphyromonas gulae LPS-induced inflammatory responses in human gingival epithelial cells. Journal of oral biosciences, 64(3), 352–358. https://doi.org/10.1016/j.job.2022.05.006
162. Yagci, F., Balkaya, H., & Demirbuga, S. (2021). Discoloration Behavior of Resin Cements Containing Different Photoinitiators. The International journal of periodontics & restorative dentistry, 41(3), e113–e120. https://doi.org/10.11607/prd.5376
163. Alonso-Español, A., Bravo, E., Carrillo de Albornoz, A., Martínez, M., Doll-Nikutta, K., Winkel, A., Stiesch, M., Herrera, D., Alonso, B., & Sanz, M. (2025). Antimicrobial Effect and Cytocompatibility After Using Different Decontamination Methods on Titanium Implant Surfaces: An In Vitro Study. Clinical oral implants research, 36(5), 626–639. https://doi.org/10.1111/clr.14410

164. Marya, Charu Mohan; Singroha, Swati; Nagpal, Ruchi; Taneja, Pratibha; Kataria, Sakshi; Kashyap, Parul .Effetto del collutorio al melograno sulla salute gengivale: Uno studio clinico randomizzato controllato di 21 giorni. Rivista dell'Associazione indiana di odontoiatria sanitaria pubblica 20(4):p 427-431, ottobre-dicembre 2022. | DOI: 10.4103/jiaphd.jiaphd_193_21
165. Dinesh, H., Sundar, S., Kannan, S., Ramadoss, R., Selvam, S. P., & Ramani, P. (2024). Synthesis and Characterization of Epigallocatechin Gallate-mediated Hydroxyapatite. Pharmaceutical nanotechnology, 12(2), 165–170. https://doi.org/10.2174/2211738511666230607113610
166. Marya, Charu Mohan; Singroha, Swati; Nagpal, Ruchi; Taneja, Pratibha; Kataria, Sakshi; Kashyap, Parul .Effetto del collutorio al melograno sulla salute gengivale: Uno studio clinico randomizzato controllato di 21 giorni. Rivista dell'Associazione indiana di odontoiatria sanitaria pubblica 20(4):p 427-431, ottobre-dicembre 2022. | DOI: 10.4103/jiaphd.jiaphd_193_21
167. Fakhri, E., Samadi Kafil, H., Naghizadeh, M., Eslami, H., & Sefidan, F. Y. (2023). Antimicrobial effect of grape seed extract as a potential intracanal medicament combined with Nd:YAG laser. Australian endodontic journal : the journal of the Australian Society of Endodontology Inc, 49 Suppl 1, 209–216. https://doi.org/10.1111/aej.12718
168. Moghadam, N. C. Z., Jasim, S. A., Ameen, F., Alotaibi, D. H., Nobre, M. A. L., Sellami, H., & Khatami, M. (2022). Nickel oxide nanoparticles synthesis using plant extract and evaluation of their antibacterial effects on Streptococcus mutans. Bioprocess and biosystems engineering, 45(7), 1201–1210. https://doi.org/10.1007/s00449-022-02736-6 (Retraction published Bioprocess Biosyst Eng. 2024 Feb;47(2):299. doi: 10.1007/s00449-024-02967-9.)
169. Delavarian, F., Ghorbanzadeh, R., & Salehi-Vaziri, A. (2023). Effects of nano-micelles curcumin-based photodynamic therapy on expression of RUNX2 as an indicator of bone regeneration in orthodontic tooth movement. Photodiagnosis and photodynamic therapy, 44, 103775. https://doi.org/10.1016/j.pdpdt.2023.103775
170. Alipour, M., Fadakar, S., Aghazadeh, M., Salehi, R., Samadi Kafil, H., Roshangar, L., Mousavi, E., & Aghazadeh, Z. (2021). Synthesis, characterization, and evaluation of curcumin-loaded endodontic reparative material. Journal of biochemical and molecular toxicology, 35(9), e22854. https://doi.org/10.1002/jbt.22854
171. Tambur, Z., Miljković-Selimović, B., Opačić, D., Vuković, B., Malešević, A., Ivančajić, L., & Aleksić, E. (2021). Inhibitory effects of propolis and essential oils on oral bacteria. Journal of infection in developing countries, 15(7), 1027–1031. https://doi.org/10.3855/jidc.14312
172. Kumar, R., Mirza, M. A., Naseef, P. P., Kuruniyan, M. S., Zakir, F., & Aggarwal, G. (2022). Exploring the Potential of Natural Product-Based Nanomedicine for Maintaining Oral Health. Molecules (Basel, Switzerland), 27(5), 1725. https://doi.org/10.3390/molecules27051725
173. Atila, D., Dalgic, A. D., Krzemińska, A., Pietrasik, J., Gendaszewska-Darmach, E., Bociaga, D., Lipinska, M., Laoutid, F., Passion, J., & Kumaravel, V. (2024). Injectable Liposome-Loaded Hydrogel Formulations with Controlled Release of Curcumin and α-Tocopherol for Dental Tissue Engineering. Advanced healthcare materials, 13(23), e2400966. https://doi.org/10.1002/adhm.202400966
174. de Araújo, L. P., Marchesin, A. R., Carpena, L. P., Gobbo, L. B., Ferreira, N. S., de Almeida, J. F. A., & Ferraz, C. C. R. (2024). Outcome of curcumin-based photodynamic therapy in endodontic microsurgery: A case report. Photodiagnosis and photodynamic therapy, 45, 103994. https://doi.org/10.1016/j.pdpdt.2024.103994
175. Luo, Y., Liu, C., Liu, J., Wang, H., Fu, Y., & Liu, Y. (2025). Exploration of Liuwei Dihuang Pill on periodontitis based on network pharmacology and molecular docking. Medicine, 104(20), e42466. https://doi.org/10.1097/MD.0000000000042466
176. Justo, M. P., Cardoso, C. B. M., Cantiga-Silva, C., de Oliveira, P. H. C., Sivieri-Araújo, G., Azuma, M. M., Ervolino, E., & Cintra, L. T. A. (2022). Curcumin reduces inflammation in rat apical periodontitis. International endodontic journal, 55(11), 1241–1251. https://doi.org/10.1111/iej.13819

177. Derman, S. H. M., Lantwin, E. M., Barbe, A. G., & Noack, M. J. (2021). Does a pretreatment with a dentine hypersensitivity mouth-rinse compensate the pain caused by professional mechanical plaque removal? A single-blind randomized controlled clinical trial. Clinical oral investigations, 25(5), 3151–3160. https://doi.org/10.1007/s00784-020-03643-4
178. Reis, M. V. P., Souza, G. L., Soares, P. B. F., Souza, M. A., Soares, C. J., & Moura, C. C. G. (2020). Effect of ScLL and 15d-PGJ2 on viability and cytokine release in LPS-stimulated fibroblasts: an in vitro study. Brazilian oral research, 34, e013. https://doi.org/10.1590/1807-3107bor-2020.vol34.0013 .
179. Cirano, F. R., Molez, A. M., Ribeiro, F. V., Tenenbaum, H. C., Casati, M. Z., Corrêa, M. G., & Pimentel, S. P. (2021). Resveratrol and insulin association reduced alveolar bone loss and produced an antioxidant effect in diabetic rats. Journal of periodontology, 92(5), 748–759. https://doi.org/10.1002/JPER.19-0718
180. Kendell-Wall, R., Nguyen, J. T., Salleras, F., Kamboj, A. S., Diwen Tan, S. A., Manish Trivedi, V., de Mello-Neto, J. M., & Rodrigues Amaral, R. (2024). Antimicrobial efficacy of Odontopaste in endodontics: a systematic review. Evidence-based dentistry, 25(3), 166. https://doi.org/10.1038/s41432-024-01000-y
181. Cunha Neto, M. A. D., Coêlho, J. A., Pinto, K. P., Cuellar, M. R. C., Marcucci, M. C., Silva, E. J. N. L., Andrade, F. B., & Sassone, L. M. (2021). Antibacterial Efficacy of Triple Antibiotic Medication With Macrogol (3Mix-MP), Traditional Triple Antibiotic Paste, Calcium Hydroxide, and Ethanol Extract of Propolis: An Intratubular Dentin Ex Vivo Confocal Laser Scanning Microscopic Study. Journal of endodontics, 47(10), 1609–1616. https://doi.org/10.1016/j.joen.2021.07.014
182. Khalil, H. F., Metwalli, N. E. E. D., Magdy, S., & Shamel, M. (2025). Tissue-friendly dentin treatments as a potential element in revascularization protocol (ex-vivo study). BMC oral health, 25(1), 184. https://doi.org/10.1186/s12903-025-05550-0
183. Zheng, X., Chen, J., Liu, J., Shi, X., Li, G., Shi, Q., Zhang, J., & Li, Y. (2024). The osteogenic effects of sappanchalcone in vitro and in vivo. Journal of periodontal research, 59(1), 84–93. https://doi.org/10.1111/jre.13189
184. Nittayananta, W., Wongwitthayakool, P., Srichana, T., Setthanurakkul, C., Yampuen, P., Terachinda, P., Deebunjerd, T., & Tachapiriyakun, J. (2023). α-Mangostin and lawsone methyl ether in tooth gel synergistically increase its antimicrobial and antibiofilm formation effects in vitro. BMC oral health, 23(1), 840. https://doi.org/10.1186/s12903-023-03511-z
185. Prado, M. M., Figueiredo, N., Pimenta, A. L., Miranda, T. S., Feres, M., Figueiredo, L. C., de Almeida, J., & Bueno-Silva, B. (2022). Recent Updates on Microbial Biofilms in Periodontitis: An Analysis of In Vitro Biofilm Models. Advances in experimental medicine and biology, 1373, 159–174. https://doi.org/10.1007/978-3-030-96881-6_8
186. Yao, L., Sadeghirad, B., Li, M., Li, J., Wang, Q., Crandon, HN, ... e Busse, JW (2023). Gestione del dolore cronico secondario a disturbi temporomandibolari: una revisione sistematica e una meta-analisi di rete di studi randomizzati. bmj , 383 .
187. Aabed, K., Moubayed, N., BinShabaib, M. S., & ALHarthi, S. S. (2022). Is a single session of antimicrobial photodynamic therapy as an adjuvant to non-surgical scaling and root planing effective in reducing periodontal inflammation and subgingival presence of Porphyromonas gingivalis and Aggregatibacter actinomycetemcomitans in patients with periodontitis?. Photodiagnosis and photodynamic therapy, 38, 102847. https://doi.org/10.1016/j.pdpdt.2022.102847
188. Blank, E., Grischke, J., Winkel, A. et al. Valutazione della colonizzazione del biofilm su impianti dentali multicomponente in un modello di ratto. BMC Oral Health 21 , 313 (2021). https://doi.org/10.1186/s12903-021-01665-2

189. Sterne, J. A. C., Savović, J., Page, M. J., Elbers, R. G., Blencowe, N. S., Boutron, I., Cates, C. J., Cheng, H.-Y., Corbett, M. S., Eldridge, S. M., Hernán, M. A., Hopewell, S., Hróbjartsson, A., Junqueira, D. R., Jüni, P., Kirkham, J. J., Lasserson, T., Li, T., McAleenan, A., Reeves, B. C., Shepperd, S., Shrier, I., Stewart, L. A., Tilling, K., White, I. R., Whiting, P. F., & Higgins, J. P. T. (2019). RoB 2: A revised tool for assessing risk of bias in randomised trials. BMJ, 366, l4898. https://doi.org/10.1136/bmj.l4898
190. Gunjal, S., Hampiholi, V., Ankola, A. V., & Pateel, D. G. S. (2024). Comparison of the effectiveness of Morus alba and chlorhexidine gels as an adjunct to scaling and root planing on stage II periodontitis - A randomized controlled clinical trial. International journal of dental hygiene, 22(3), 717–726. https://doi.org/10.1111/idh.12781
191. Sundaram, G., Theagarajan, R., Murthy, G. D., & Kanimozhi, G. (2021). Effect of piper extract mouthwash as postprocedural rinse on levels of Porphyromonas gingivalis in periodontitis patients. Journal of Indian Society of Periodontology, 25(5), 418–421. https://doi.org/10.4103/jisp.jisp_509_20
192. Siddharth, M., Singh, P., Gupta, R., Sinha, A., Shree, S., & Sharma, K. (2020). A Comparative Evaluation of Subgingivally Delivered 2% Curcumin and 0.2% Chlorhexidine Gel Adjunctive to Scaling and Root Planing in Chronic Periodontitis. The journal of contemporary dental practice, 21(5), 494–499.
193. Agarwal, A., & Chaudhary, B. (2020). Clinical and microbiological effects of 1% Matricaria chamomilla mouth rinse on chronic periodontitis: A double-blind randomized placebo controlled trial. Journal of Indian Society of Periodontology, 24(4), 354–361. https://doi.org/10.4103/jisp.jisp_441_19
194. Dolly, A. S., Shankar, P. L. R., Saravanan, A. V., Pandian, K. R., Sindhujaa, R., & Rashik, K. M. M. (2024). Subgingivally Delivered Spirulina Gel and Chlorhexidine Gel in Periodontitis Patients - A Comparative Study. Indian journal of dental research : official publication of Indian Society for Dental Research, 35(4), 406–411. https://doi.org/10.4103/ijdr.ijdr_271_24
195. Scribante, A., Gallo, S., Pascadopoli, M., Frani, M., & Butera, A. (2024). Ozonized gels vs chlorhexidine in non-surgical periodontal treatment: A randomized clinical trial. Oral diseases, 30(6), 3993–4000. https://doi.org/10.1111/odi.14829
196. Waqar, S. M., Razi, A., Qureshi, S. S., Saher, F., Zaidi, S. J. A., & Kumar, C. (2024). Comparative evaluation of propolis mouthwash with 0.2% chlorhexidine mouthwash as an adjunct to mechanical therapy in improving the periodontitis among perimenopausal women: a randomized controlled trial. BMC oral health, 24(1), 26. https://doi.org/10.1186/s12903-023-03768-4
197. Seth, T. A., Kale, T. A., Lendhey, S. S., & Bhalerao, P. V. (2022). Comparative evaluation of subgingival irrigation with propolis extract versus chlorhexidine as an adjunct to scaling and root planing for the treatment of chronic periodontitis: A randomized controlled trial. Journal of Indian Society of Periodontology, 26(2), 151–156. https://doi.org/10.4103/jisp.jisp_613_20
198. Guru, S. R., Reddy, K. A., Rao, R. J., Padmanabhan, S., Guru, R., & Srinivasa, T. S. (2020). Comparative evaluation of 2% turmeric extract with nanocarrier and 1% chlorhexidine gel as an adjunct to scaling and root planing in patients with chronic periodontitis: A pilot randomized controlled clinical trial. Journal of Indian Society of Periodontology, 24(3), 244–252. https://doi.org/10.4103/jisp.jisp_207_19
199. Rathod, A., Jaiswal, P., Kale, B., & Masurkar, D. (2023). Comparative Evaluation of the Effectiveness of Triphala and Chlorhexidine in Full-mouth Disinfection Treatment of Periodontitis in Type 2 Diabetes Patients. The journal of contemporary dental practice, 24(10), 798–801. https://doi.org/10.5005/jp-journals-10024-3573
200. Chawla, R., Patil, A., Mistry, V. D., Waghmare, A. S., Ronad, S., & Deshmukh, C. V. (2024). A novel era of probiotics in dentistry: A double-blind randomised clinical trial. Journal of Indian Association of Public Health Dentistry, 22(1), 45–49. https://doi.org/10.4103/jiaphd.jiaphd_142_23

201. Basudan, A. M., Al-Zawawi, A. S., Divakar, D. D., Shaheen, M. Y., & Aldulaijan, H. A. (2023). Efficacy of 0.12% Chlorhexidine and Salvadora persica-based Mouthwash in Reducing Oral Candida Carriage and Periodontal Inflammation in Cigarette Smokers and Non-smokers after Non-surgical Periodontal Therapy. Oral health & preventive dentistry, 21, 219–228. https://doi.org/10.3290/j.ohpd.b4169713
202. Sanghavi, A., Shettigar, L., Chopra, A., Shah, A., Lobo, R., Shenoy, P. A., Gadag, S., Nayak, U. Y., Shravya S, M., Kamath, S. U., & Nayak, P. P. (2024). Efficacy of Lycium barbarum (Goji berry) mouthwash for managing periodontitis: a randomized clinical trial. F1000Research, 12, 302. https://doi.org/10.12688/f1000research.129891.4
203. National Heart, Lung, and Blood Institute. Study Quality Assessment Tool. Available online: https://www.nhlbi.nih.gov/health-topics/study-quality-assessment-tools.
